# A novel internal reference microorganism-based method reveals wild-enriched *Penicillium* for enhancing growth and disease resistance in *Fritillaria thunbergii*

**DOI:** 10.3389/fmicb.2026.1746815

**Published:** 2026-02-11

**Authors:** Long Cai, Jian Sun, Haishen Li, Chuanbao Wang, Minda Zhang, Qingsong Shao, Zhian Wang

**Affiliations:** 1 Zhejiang Research Institute of Traditional Chinese Medicine Co., Ltd., Hangzhou, China; 2 State Key Laboratory for Quality Ensurance and Sustainable Use of Dao-di Herbs, Beijing, China; 3 Zhejiang Provincial Key Laboratory of Resources Protection and Innovation of Traditional Chinese Medicine, Zhejiang Agriculture and Forestry University, Hangzhou, China

**Keywords:** biological control, *Fritillaria thunbergii* Miq., internal reference microorganism, microbiome rewilding, *Penicillium* sp., plant domestication, rhizosphere microbiome

## Abstract

**Introduction:**

*Fritillaria thunbergii Miq.* is an important traditional Chinese medicinal herb, but bulb rot disease causes severe losses during its cultivation. Screening and reintroducing beneficial microorganisms from the wild rhizosphere is an effective strategy to increase disease resistance in cultivated plants. However, the absolute quantitative characteristics of microorganisms cannot be reflected by traditional methods based on relative abundance analysis due to compositional bias.

**Methods:**

In this study, we identified Clavatospora as a host-specific internal reference microorganism (IRM) and established an IRM-based relative abundance differential microorganism analysis (IRMRADMA) method.

**Results:**

Application of this method revealed that wild *F. thunbergii* possesses greater potential than cultivated plants for mining beneficial rhizosphere microorganisms. Specifically, *Penicillium* was identified as a key wild-enriched genus. Subsequently, two strains, *Penicillium korosum* and *Penicillium aculeatum*, were isolated from the rhizosphere of wild *F. thunbergii*. Functional tests confirmed that these strains demonstrated dual functions in disease suppression and plant growth promotion by solubilizing phosphate, producing siderophores, antagonizing pathogens, and upregulating defenserelated genes.

**Conclusion:**

This study established the IRM-RADMA method and identified key *Penicillium* strains from the wild *Fritillaria thunbergii* rhizosphere. Their confirmed dual functions in growth promotion and disease suppression validate the ‘plant microbiome rewilding’ strategy, offering a new paradigm for the biological control of *F. thunbergii* diseases.

## 1 Introduction

*Fritillaria thunbergii* Miq., a perennial herb of the Liliaceae family, is valued for its ability to clear heat and resolve phlegm to relieve cough. It is mainly distributed in Ningbo, Jinhua, and the other regions of Zhejiang Province, with a cultivation area of approximately 3,333 ha, yielding 10,000 tons annually and generating an output value nearing USD 300 million. Wild *F. thunbergii* resources are gradually being depleted, with only a sparse distribution in the mountainous areas of northern Zhejiang.

Current cultivation practices for *F. thunbergii* involve high-density planting, excessive irrigation and fertilization, and widespread pesticide use. Although cultivated *F. thunbergii* yields higher than wild *F. thunbergii*, long-term domestication has led to “domestication syndrome,” characterized by significantly weakened disease resistance and the frequent occurrence of various diseases, including bulb rot, with a field incidence exceeding 22%. Cultivation alters plant rhizosphere microbial communities, and some taxa within these communities are crucial for promoting plant growth and enhancing resistance (Yue et al., 2023; Barnes et al., 2025). In *Fritillaria* research, Liu et al. (2023) reported that with increasing cultivation time of *Fritillaria unibracteata*, the abundance of *Pseudomonas* in its rhizosphere decreased, and its pathogen-suppressing function gradually weakened; Zhou et al. (2022) reported similar phenomena in *Fritillaria taipaiensis*.

The domestication process from wild to cultivated also significantly altered the plant rhizosphere microbial structure. Studies have shown that wild plant rhizosphere soil contains abundant potentially beneficial microorganisms; reintroducing these microbial taxa from wild plants into cultivated habitats can significantly promote growth or enhance disease resistance in cultivated varieties, representing an effective solution to the “domestication syndrome” and to the obstacles posed by continuous cropping (Porter and Sachs, 2020; Kong and Liu, 2022; Raaijmakers and Kiers, 2022). Cao X. et al. (2025) isolated *Burkholderia* from the rhizosphere of wild *Coptis chinensis*, which exhibited dual growth-promoting and disease-suppressing mechanisms; colonizing the cultivated *C. chinensis* rhizosphere with this strain significantly increased disease resistance and growth. Chang et al. (2025) transplanted wild rice rhizosphere microbial suspensions into soil and reported that they increased soil nitrogenase activity and promoted rice growth. These studies provide necessary theoretical support for plant microbiome rewilding. However, the prerequisite for plant microbiome rewilding is mining and isolating beneficial microbial taxa from wild plant rhizospheres, followed by reintroduction into cultivated habitats. Currently, research on the isolation and utilization of beneficial microorganisms from the rhizosphere of wild *F. thunbergii* remains entirely unexplored.

High-throughput sequencing technologies (e.g., ITS amplicon sequencing) have become central to rhizosphere microbiome research, where accurate screening of target microorganisms is a key prerequisite for deciphering their functional mechanisms. Traditional primary abundance-based differential microorganism analysis (PA-DMA) relies primarily on statistical tests such as the Wilcoxon test and the Kruskal-Wallis test (Wu et al., 2025). However, these computations are generally based on normalized relative abundance, which implicitly assumes a constant total microbial load across samples (Gloor et al., 2017). In reality, abiotic factors, such as soil properties and regional climate, significantly influence the total microbial load. Concurrently, microbiome data based on relative abundance exhibit compositional closure: the sum of all microbial taxa’s relative abundances invariably equals 1 or 100% (Quinn et al., 2018). Consequently, an increase in one taxon’s relative abundance necessitates a decrease in others, making it impossible to determine whether an observed rise stems from the genuine growth of that taxon or the decline of others (StämmLer et al., 2016). This property may cause taxa with identical absolute abundances to show significant relative abundance differences after normalization, leading to misinterpretation of differential significance and thereby increasing the risk of false negatives and false positives (Mandal et al., 2015; Morton et al., 2019). Thus, the core limitation of PA-DMA is its inability to reveal true intersample variations in total microbial load, compromising the accuracy of differential microbe screening (Guo et al., 2020). This dilemma fundamentally arises from the absence of stably expressed internal reference standards across samples, preventing relative abundance data from reflecting absolute differences authentically.

To overcome the limitations of relative abundance-based differential microbe screening, this study employed an innovative strategy: rhizosphere soils of *F. thunbergii* and its co-located companion plants (*Lycoris radiata, Brassica pekinensis*) were collected to construct three location-matched sample sets for ITS amplicon sequencing. Using microbial presence/absence as the screening criterion, we removed microorganisms shared between *F. thunbergii* and companion plants while retaining core fungi unique to multilocation *F. thunbergii*, thereby identifying the closely associated fungal genus as the IRM. The relative abundance of this fungal genus subsequently served as a cross-sample normalization internal control to recalculate the IRM-normalized quantity of other microorganisms for rescreening differential taxa, and the differential taxa screened by the two methods were compared.

This study aims to achieve three core objectives: first, to elucidate the evolutionary trajectory of rhizosphere fungal communities—particularly beneficial microorganisms—during the domestication of *F. thunbergii* from wild to cultivated forms; second, to establish an optimized method for screening differential microorganisms; and third, to isolate and characterize key beneficial microorganisms from wild *F. thunbergii* and clarify their functional mechanisms.

We propose the following specific hypotheses: (1) The rhizosphere mycobiome of wild *F. thunbergii* exhibits greater functional diversity and a higher abundance of beneficial fungal guilds than that of their cultivated counterparts; (2) Employing a plant-specific IRM exclusively associated with *F. thunbergii* will alleviate the limitations of traditional relative abundance-based differential analysis, thereby enhancing the detection accuracy of ecologically relevant differential fungal taxa; (3) Wild-enriched fungal genera identified using the IRM-anchored differential approach will include strains with practical functional potential—especially in disease suppression and plant growth promotion—that may serve as candidate beneficial microorganisms for the sustainable cultivation of *F. thunbergii*. Overall, this study lays a theoretical foundation and provides microbial resources for the screening of beneficial microbes from wild *F. thunbergii* and the development of biocontrol strategies against diseases in cultivated *F. thunbergii*.

## 2 Materials and methods

### 2.1 Sampling sites and methods

According to the methodologies described by Fang et al. (2022) and Liu et al. (2023), with modifications, rhizosphere soils of cultivated *F. thunbergii* were collected from two main production areas: Haishu (HFt) and Pan’an (PFt). Six random 3 × 3 m^2^ plots (numbered 1–6) were selected per location. Five healthy plants per plot were sampled as a single biological replicate using a *Z*-shaped sampling pattern. At 20 cm from the soil surface, sterile stainless-steel shovels were used to collect the soil (20–30 cm depth). Soils from five plants per plot were sieved (2 mm), mixed, and stored in sterile cryotubes at −80°C for ITS amplicon sequencing. Wild *F. thunbergii* rhizosphere soils (WFt) were collected from Changxing County: three wild plants within 25 m^2^ constituted one biological replicate (six replicates total). Sampling followed cultivated *F. thunbergii* protocols, with samples stored at −80°C. The companion plant rhizosphere soils within a 30 cm linear distance were collected as controls via identical methods. In this study, “Companion plants” refers to plant species adjacent to *F. thunbergii* within the same plot that share growth environments (soil, air, and water) without spatial overlap. The three groups were as follows: wild *F. thunbergii*–*Lycoris radiata* (WFt-Lr), Haishu *F. thunbergii*-*Brassica pekinensis* (HFt-HBp), and Pan’an *F. thunbergii*-*Brassica pekinensis* (PFt-PBp). The sampling sites, methods, and partial soil physical and chemical properties are detailed in Supplementary Figure S1 and Supplementary Table S1.

### 2.2 DNA extraction and PCR amplification

DNA extraction and PCR amplification were performed following the methods of Ping et al. (2024) with slight modifications. Microbial genomic DNA was extracted from 0.25 g soil samples via the Rhizosphere Soil DNA Kit (TIANGEN, GER) according to the manufacturer’s protocols. DNA integrity was confirmed by 1% agarose gel electrophoresis, and DNA quality was quantified via a NanoDrop 2000 UV-Vis spectrophotometer (Thermo Scientific, Wilmington, United States). PCR amplification was conducted with the universal ITS primers ITS1 and ITS4 (Siddiqui et al., 2022; Supplementary Table S2). The PCR products were purified with AMPure XT beads (Beckman Coulter Genomics, Danvers, MA, United States), quantified with a Qubit (Invitrogen, United States), and verified via 2% agarose gel electrophoresis. Purified amplicons were recovered via an AMPure XT bead recovery kit. The recovered products were assessed via an Agilent 2100 Bioanalyzer (Agilent, United States) and quantified via an Illumina Library Quantification Kit (Kapa Biosciences, Woburn, MA, United States). The libraries were sequenced on NovaSeq PE250 platform.

### 2.3 Sequencing data processing and analysis

Samples were sequenced on an Illumina NovaSeq platform according to the manufacturer’s recommendations, provided by LC-Bio. For the paired-end sequencing data, demultiplexing was first performed based on barcode information to assign reads to samples, followed by the removal of adapter and barcode sequences. The primer sequences and barcode were trimmed from RawData. Each pair of paired-end reads was merged into a single longer tag based on overlapping regions. A sliding-window quality scan (default window size: 100 bp) was applied to the sequencing reads. Sequences with > 5% ambiguous bases (N) after truncation and chimeric sequences were removed (Karlsson et al., 2014). DADA2 was invoked in QIIME for length filtering and denoising (Callahan et al., 2016), resulting in Amplicon Sequence Variants (ASV) feature sequences (Blaxter et al., 2005). Alpha diversity and beta diversity analyses were also performed. Taxonomic annotation was conducted. The abundance of each fungal genus in each sample was calculated based on both the RDP and UNITE databases and the ASV (feature) abundance table. Differential fungal genera analysis for *F. thunbergii* was performed via the Wilcoxon rank-sum test (two sites), the Kruskal-Wallis test (three sites) and visualized by Volcano plot (relative abundance > 0.005% in at least one group, *p* < 0.05). STAMP is also used for differential analysis (Parks et al., 2014). Community function prediction was performed at the ASV level using FUNGuild and PICRUSt2.

Co-occurrence network analysis was performed for fungal taxa with a relative abundance > 0.01% in at least one sample. Gephi v0.9.3 was used to visualize co-occurrence networks and calculate topological features of fungal networks (Cao X. et al., 2025).

Linear discriminant analysis (LDA) effect size (LEfSe) was applied to determine the features (differentially enriched microbial taxa and functions) most likely to explain differences between wild and cultivated *F. thunbergii*. Taxa with an LDA effect size greater than 4.5 (*P* < 0.05) were considered significant (Wang W. et al., 2024).

### 2.4 Screening of differential microorganisms via the IRMRA-DMA method

The screening of IRM for *F. thunbergii* based on the presence or absence of microorganisms by *F. thunbergii* statistical analysis was performed separately on the rhizosphere fungal genera WFt and Lr, HFt and HBp, PFt and PBp. For each production area, fungal genera shared between *F. thunbergii* and its companion plant were excluded, and *F. thunbergii*-unique fungal genera were retained. Further cross-analysis of *F. thunbergii*-unique genera from all three areas identified genera common to all three locations, defined as closely associated fungal genera (IRM).

The recalculation of relative community quantity (RCQ) for other fungal genera was calculated as follows: RCQ = relative abundance of a fungal genus/relative abundance of the IRM. For IRM with zero relative abundance in a specific group, an abundance-dependent pseudo-count was applied: adjusted abundance = 0.5* detection limit. The detection limit of relative abundance in this amplicon dataset was 0.002%, thus, the pseudo-count = 0.001%. For other fungal genera with a relative abundance of 0 in a specific group, they were still treated as 0.

The differential fungal genus screening based on the IRMRA-DMA analysis for *F. thunbergii* was conducted via the Wilcoxon rank-sum test (comparisons between two sites) and the Kruskal-Wallis test (comparisons across three sites).

### 2.5 Isolation and identification of rhizosphere fungi

Fungal isolation from the soil was performed using the gradient dilution plate method (Liu B. et al., 2020). One gram of selected rhizosphere soil samples from section 1.1 was weighed and serially diluted to concentrations of 10^–3^, 10^–4^, 10^–5^, and 10^–6^. 200 μL of each soil suspension were spread evenly onto PDA plates containing 0.01% chloramphenicol. The plates were incubated at 28°C. After 3–5 days, distinct colonies were picked with an inoculation needle and transferred to fresh PDA plates. After 3–5 days of additional incubation to obtain pure colonies, colony and spore characteristics were observed. Fungal DNA extraction was subsequently performed. Mycelial DNA was amplified via the universal fungal ITS primers ITS1 and ITS4. The PCR products were electrophoresed on 1.5% agarose gels (120 V, 60 min). Amplified fragments were purified and sequenced. ITS2 sequences of strains with high homology were selected as references. Multiple sequence alignment was performed using MEGA 7.0. Phylogenetic trees were constructed via the neighbor-joining method. Node support was assessed via bootstrap analysis with 1,000 replicates.

### 2.6 Growth promotion traits analysis of WFt-032 and WFt-056

For the detection of phosphate solubilization ability, it was analyzed according to Doilom et al. (2020). Purified strains were inoculated centrally on inorganic phosphorus media and incubated inverted at 28°C for 5 days. The formation of a transparent halo around the colonies indicated the fungi’s ability to solubilize phosphate. The solubilization index (SI) was calculated as SI = D/d, where D = the diameter of the phosphate solubilization halo and d = the colony diameter.

Also the detection of siderophore production was done following the methodologies described by Sahu et al. (2023). Purified strains were inoculated centrally on CAS detection media and incubated inverted at 28°C for 5 days. The formation of an orange-red halo around the colonies indicated the ability of the fungi to produce siderophores. The siderophore production index (SPI) was calculated as SPI = M/m, where M = the diameter of the orange-red halo and m = the colony diameter.

### 2.7 Analysis of antagonistic activity of WFt-032 and WFt-056 against bulb rot pathogens

Pathogens *Fusarium oxysporum* and *Alternaria tenuissima* were cultured on PDA at 28°C in darkness for 5 days, and samples were collected using a 5 mm cork borer at colony edges. A 5-mm pathogen plug was placed at the center of a fresh PDA plate. Strains WFt-032 and WFt-056 were inoculated symmetrically 2 cm from the plate edge. Sterile PDA plugs served as controls. After 7 days of dual culture at 28°C in darkness, the inhibition efficiency (IE%) was calculated as follows: IE (%) = (1 − Rc/Rs) × 100, where Rc = radius of pathogen colony toward the antagonist and Rs = radius in solo culture. The experiments were performed in triplicate (Zhao et al., 2021).

WFt-032 and WFt-056 were cultured on PDA for 5 days until sporulation. The spore suspensions were prepared in sterile water and adjusted to 1 × 10^6^ CFU/mL employing a hemocytometer. One milliliter of suspension was inoculated into 100 mL PDB and incubated at 28°C and 220 r/min for 3 days. The fermentation broth was filtered (0.22 μm) and mixed with PDA at a ratio of 1:10 (v/v). PDA with sterile water served as a control. A 5-mm plug of *F. oxysporum* or *A. tenuissima* was inoculated centrally. After 7 days at 28°C in darkness, the inhibition rate (IR%) was calculated as follows: IR (%) = (1 − Dcf/Dck) × 100, where Dcf = colony radius in culture filtrate-treated PDA and Dck = colony radius in control PDA.

### 2.8 Effects of WFt-032 and WFt-056 on bulb disease resistance and resistance gene expression in *F. thunbergii*

The following methods were used as described by Zhao et al. (2021). At first the preparation of fermentation filtrates for WFt-032, WFt-056 and pathogen spore suspension strains were cultured on PDA at 28°C for 5 days until sporulation. The spore suspensions were adjusted to 1 × 10^6^ CFU/mL in sterile water employing a hemocytometer. One milliliter was inoculated into 100 mL of PDB (Zhao et al., 2020) and incubated at 28 °C and 220 r/min for 3 days. The fermentation broth was filtered (0.22 μm) and diluted 10-fold with sterile water to obtain the fungal fermentation filtrate. *F. oxysporum* and *A. tenuissima* were cultured on PDA for 5 days. Spore suspensions (1 × 10^6^ CFU/mL) of both pathogens were mixed. Second, stored bulbs of *F. thunbergii* were selected for the experiment, with 6 treatments designated as follows: G1: bulbs were immersed in WFt-032 fermentation filtrates for 6 h and air-dried, placed in germination boxes, and then 5 mL of the pathogen spore suspension was sprayed onto the bulbs; G2: bulbs were immersed in WFt-056 fermentation filtrates for 6 h and air-dried, placed in germination boxes, and then 5 mL of the pathogen spore suspension was sprayed onto the bulbs; G3: bulbs were immersed in PDB for 6 h and air-dried, placed in germination boxes, and then 5 mL of the pathogen spore suspension was sprayed onto the bulbs; finally, G4, G5, and G6 corresponded to replacing the pathogen spore suspension in G1, G2, and G3 with sterile water, respectively. The other treatments remaining the same; each of the 6 treatments included 3 bulbs with 3 replicates and all bulbs were cultured at 25°C under moisture-retaining conditions, photographs were taken when the bulbs in group G3 were completely rotten, and the disease occurrence in other groups was recorded.

Finally, resistance gene expression analysis was done considering the bulbs were sampled at three time points: immediately after fermentation filtrate treatment (0 h), 24 h post pathogen spore suspension treatment (24 h), and 48 h post pathogen spore suspension treatment (48 h). Samples were flash-frozen in liquid nitrogen for RNA extraction. The expression levels of *PR1, PR2* were analyzed. The qRT-PCR primers were designed via Premier 5.0 (Supplementary Table S2). β-actin served as an internal control. Finally, the data analysis was performed via the 2^–ΔΔCT^ method.

## 3 Results

### 3.1 Rhizosphere community diversity of fungi between wild and cultivated *F. thunbergii*

**FIGURE 1:**
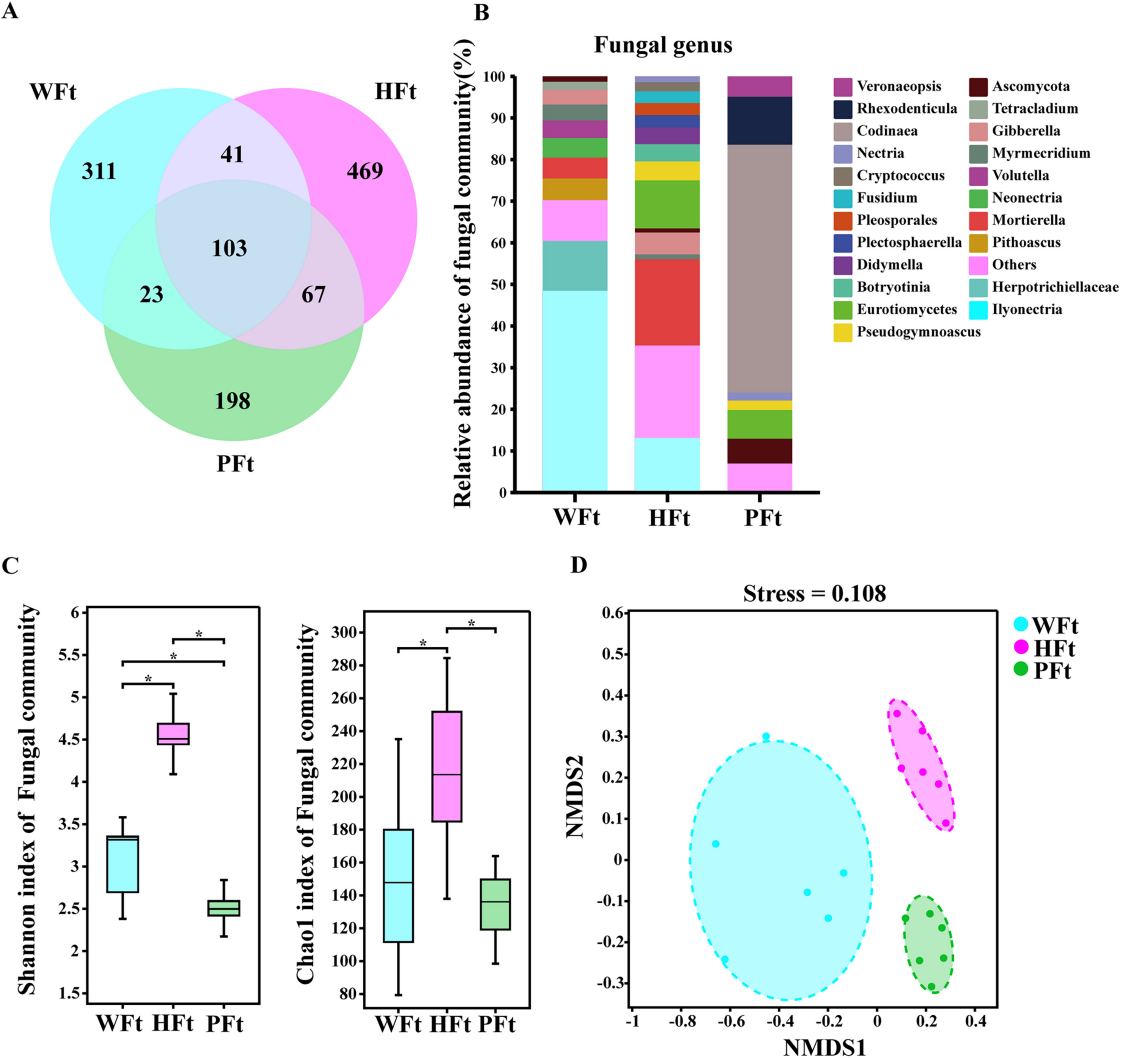
Composition and assembly of rhizomicrobiomes in wild (WFt) and cultivated (HFt, PFt) *F. thunbergii*. **(A)** Venn diagram of the familiar and unique ASVs among the rhizosphere soils of *F. thunbergii* at different sampling sites. **(B)** Composition of fungal genera in all samples of wild and cultivated *F. thunbergii*. **(C)** Shannon diversity and Chao richness indices of fungal communities in the rhizosphere of *F. thunbergii*. Statistically significant differences were determined by the Wilcoxon test (*P* < 0.05). **(D)** Nonmetric multidimensional scaling (NMDS) of the fungal microbiomes in the rhizosphere samples was performed based on the Bray-Curtis distance (*n* = 18).

Analysis of ITS amplicon samples from rhizosphere soils of *F. thunbergii* across three locations yielded 2,694,620 fungal ITS2 region sequences. Sequence data are available in the NCBI SRA under accession code PRJNA1295928. After quality control and clustering of the raw sequences using the DADA2 algorithm, 2,323 fungal amplicon sequence variants (ASVs) were obtained (Supplementary Table S3). The number of ASVs shared by *F. thunbergii* from all three locations was 103. Among the location-specific ASVs, HFt had 469 unique ASVs, exceeding WFt (311) and PFt (198), demonstrating the unique compositional features of the rhizosphere fungal communities at this site (Figure 1A). ASVs were annotated against the RDP classifier and UNITE fungal database and assigned to 12 phyla, 43 classes, 102 orders, 217 families, 416 genera, and 696 species. At the genus level, communities showed dominant taxa characteristics: *Ilyonectria, Codinaea*, *Mortierella* presented > 10% mean relative abundances (Figure 1B). *Codinaea* was exclusively detected in PFt, indicating unique distribution patterns. Alpha diversity analysis (Shannon and Chao1 indices) revealed that HFt > WFt > PFt. The HFt samples presented significantly greater fungal genus richness than the WFt and PFt samples did (Wilcoxon test, *P* < 0.05) (Figure 1C). While the Shannon index of WFt was significantly greater than that of PFt (Wilcoxon test, *P* < 0.05), no significant difference was detected in the Chao1 index (Wilcoxon test, *P* > 0.05). NMDS based on the Bray-Curtis distance matrix indicated significant divergence in fungal communities between the wild and cultivated rhizospheres (stress = 0.108) (Figure 1D), demonstrating that cultivation substantially altered the microbial composition of *F. thunbergii* rhizosphere soils.

### 3.2 Rhizosphere microbial communities of wild *F. thunbergii* show increased functional diversity

**FIGURE 2:**
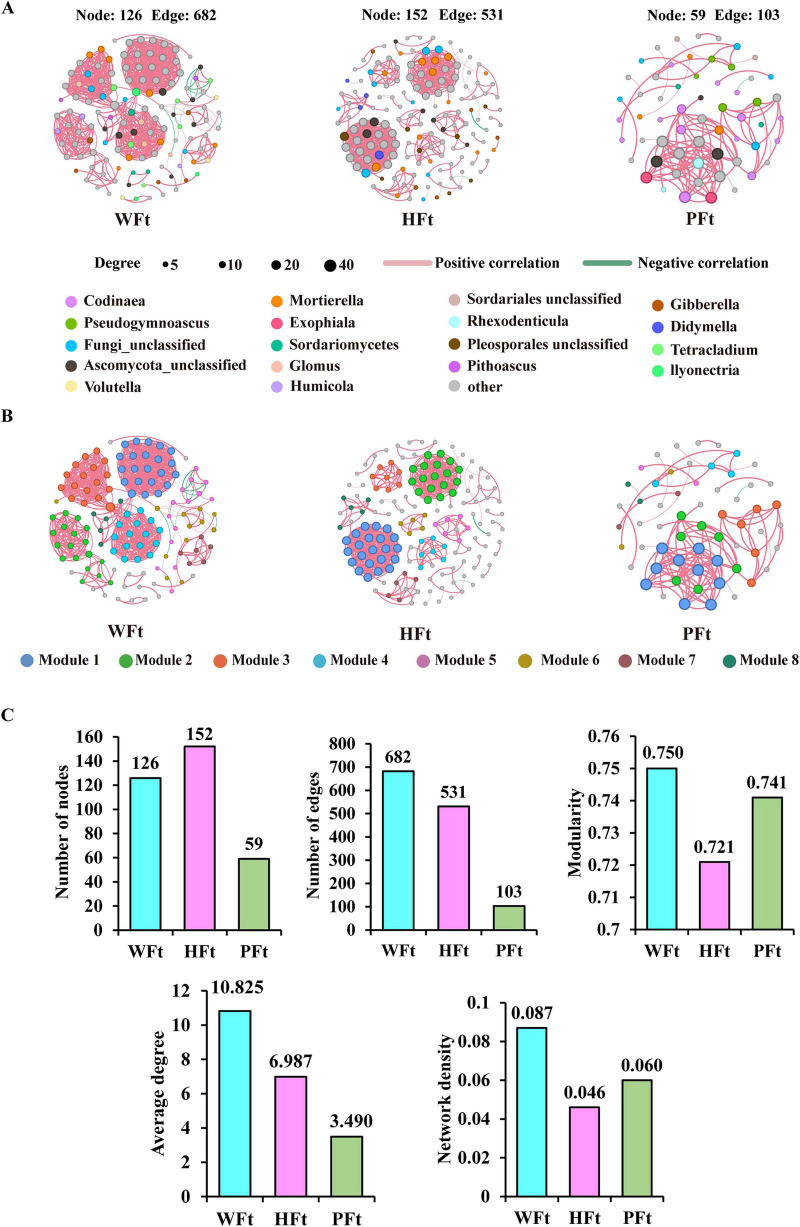
Rhizosphere fungal co-occurrence networks and topological features. **(A)** Co-occurrence networks in wild and cultivated *F. thunbergii*. Node size indicates the degree of connection. The edge color represents positive (pink) and negative (green) correlations. The thickness of each link line is proportional to the correlation coefficients of the connections. **(B)** Module distribution in wild and cultivated *F. thunbergii*. **(C)** Number of nodes, number of edges, modularity, average degree, and network density in wild and cultivated *F. thunbergii.*

The rhizosphere fungal network diagrams of WFt, HFt, and PFt exhibited typical modular characteristics, but the structures differed between cultivated and wild habitats: HFt had 152 nodes, which was higher than WFt’s 126 but only 531 edges, which were fewer than WFt’s 682, indicating closer and more frequent interactions among microbial taxa in WFt. PFt had only 59 nodes and 103 edges, which were significantly lower than WFt and HFt (Figures 2A,C and Supplementary Table S4). The dominant nodes in the co-occurrence networks differed across locations: WFt included *Mortierella*, *Tetracladium*, *Gibberella*, *Glomus*, *Pithoascus*, *Ilyonectria*, and *Volutella*; HFt included *Mortierella*, *Gibberella*, and *Fusarium*; and PFt included *Codinaea* and *Pseudogymnoascus.* These geographic differences in the core nodes demonstrated profound environmental filtering and shaping of the rhizosphere fungal assembly and interaction networks.

WFt exhibited higher modularity, average degree, and network density than did HFt and PFt, indicating more diverse functional modules. In modularity analysis, WFt fungi were evenly distributed across 6 modules (9.52–17.46% of total nodes per module), indicating greater functional diversity; in contrast, HFt and PFt contained the most nodes and edges within 2 main modules (Figure 2B), indicating functional homogenization. These results reveal differences in rhizosphere fungal network structures across habitats, with greater complexity and modular dispersion in wild habitats, likely representing key ecological strategies for environmental adaptation.

### 3.3 Functional prediction and analysis of rhizosphere fungal communities in wild and cultivated *F. thunbergii*

**FIGURE 3:**
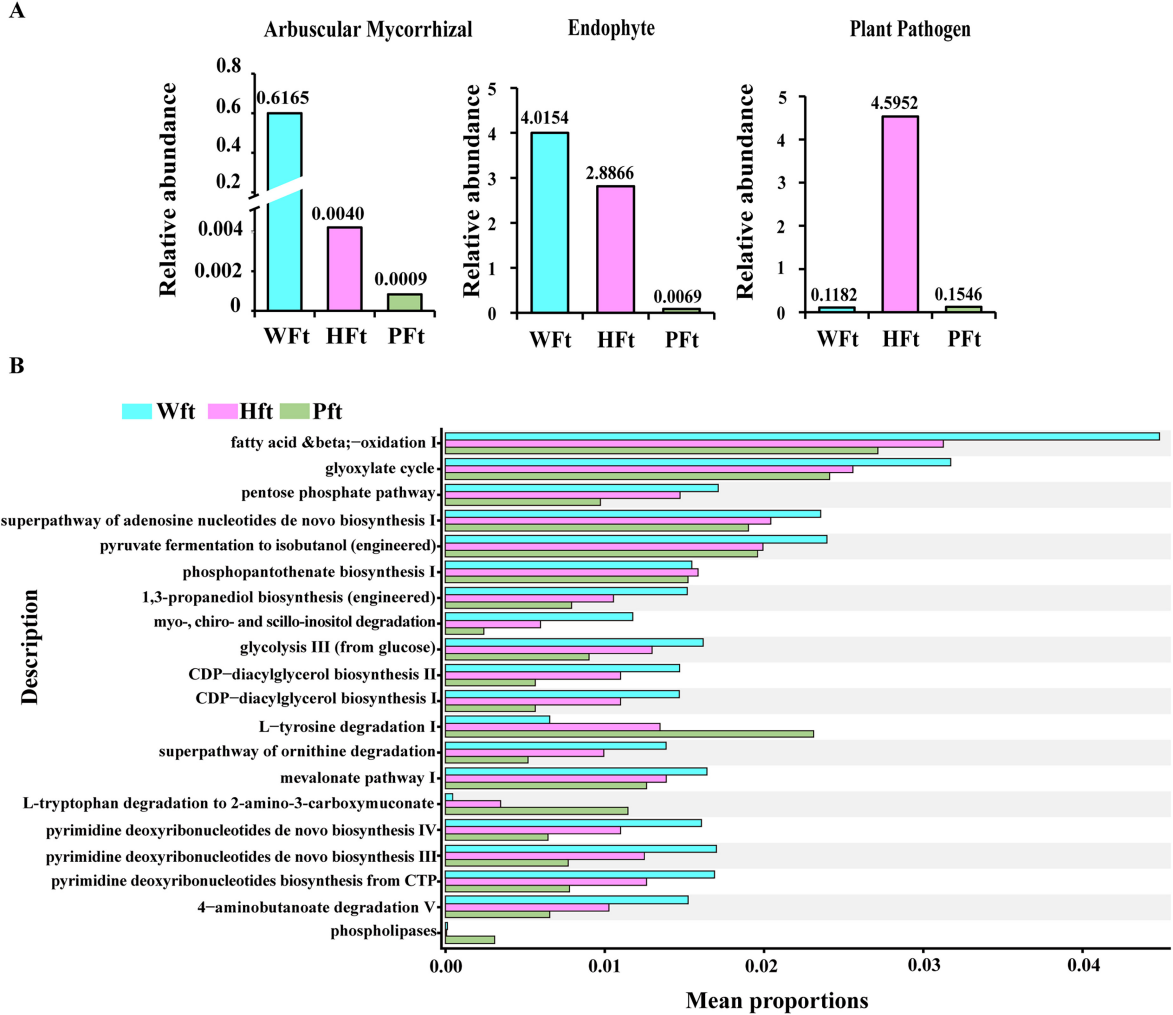
Functional prediction and analysis of rhizosphere fungal communities in wild and cultivated *F. thunbergii.* **(A)** The relative abundance of potentially beneficial and pathogenic taxa based on the FUNGuild functional annotation of fungal communities in the rhizosphere of wild and cultivated *F. thunbergii*. **(B)** PICRUSt2 functional annotation of fungal communities between wild and cultivated *F. thunbergii*.

Compared with WFt, FUNGuild functional annotation of rhizosphere fungal communities across three *F. thunbergii* habitats revealed that cultivated plants had substantially lower abundances of beneficial taxa, such as arbuscular mycorrhizae and endophytes. The percentage of arbuscular mycorrhizae decreased from 0.6165% to 0.0040% and 0.0009%, whereas that of endophytes decreased from 4.0154% to 2.8866% and 0.0069%. Conversely, potentially phytopathogenic plant pathogens significantly increased in cultivated rhizospheres, increasing from 0.1182% in WFt to 4.5952% and 0.1546% (Figure 3A). These results indicate a dual trend in cultivated *F. thunbergii*: a decline in beneficial functional groups (arbuscular mycorrhizae, endophytes) and an enrichment of potential pathogens (plant pathogens), suggesting that cultivation practices disrupt the fungal functional balance by altering the soil microecology. PICRUSt2 functional annotation also showed that the functions of rhizosphere fungi of wild and cultivated *F. thunbergii* exhibited difference (Figure 3B).

### 3.4 Screening and analysis of key rhizospheric microorganisms in wild *F. thunbergii* via PA-DMA

**FIGURE 4:**
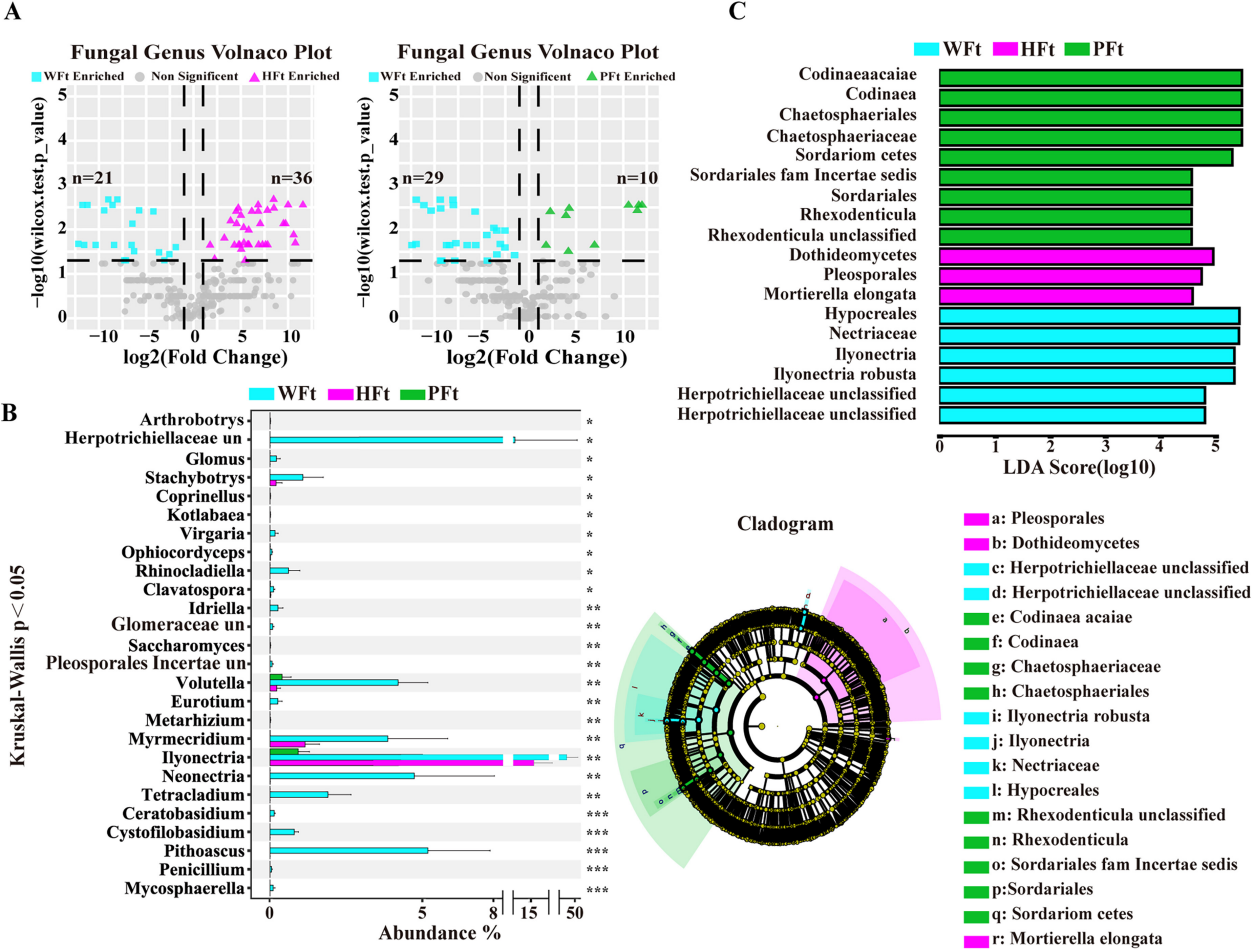
Identification of differential fungal genera in wild and cultivated *F. thunbergii*. **(A)** Volcano plot showing the differences in fungal genera between wild and cultivated *F. thunbergii* from two areas (only considering fungal genera with relative abundances > 0.005%, Wilcoxon test, *P* < 0.05). **(B)** Differential fungal genera between wild and cultivated *F. thunbergii* based on the Kruskal-Wallis test (only considering fungal genera with abundance in WFt higher than that in HFt and PFt, and with relative abundance > 0.005%, *P < 0.05, **P < 0.01, ***P < 0.001, “un” indicates “unclassified“) were considered. **(C)** Phylogenetic tree and LDA value distribution histogram analysis of species between wild and cultivated *F. thunbergii* (only considering fungal genera with relative abundances > 0.005% and LDA > 4.5).

Given that the rhizosphere of wild *F. thunbergii* harbors more fungal taxa with critical ecological functions, this study aimed to precisely identify core fungal taxa in wild and cultivated rhizospheres, particularly highly abundant groups in wild *F. thunbergii* (WFt). Fungal genus-level volcano plots (Figure 4A) revealed that, compared with HFt, WFt had 21 significantly upregulated and 36 downregulated genera (Supplementary Table S5; Wilcoxon test, *P* < 0.05), with upregulated taxa accounting for 36.84% of the differential genera; compared with PFt, WFt had 29 upregulated and 10 downregulated genera (Supplementary Table S6; Wilcoxon test, *P* < 0.05), with 74.36% upregulated taxa. Among these genera, 19 were consistently upregulated in WFt vs. both cultivated sites, including known growth-promoting taxa (*Penicillium, Glomus, Ceratobasidium*), whereas the cultivated groups were enriched in pathogenic genera such as Fusarium. STAMP-based differential expression analysis revealed consistent results (Supplementary Figure S2), indicating that environmental factors significantly influence rhizosphere fungal distribution.

The Kruskal-Wallis test identified 78 significantly differentially abundant genera across the three sites (Supplementary Table S7). Twenty-six genera presented significantly greater relative abundances in the wild samples than in the samples from both cultivation sites (Figure 4B). A cross-comparison of these 26 genera with the 19 previously identified differential genera revealed the complete inclusion of the 19 genera. Thus, these 26 genera were defined as “wild-enriched high-abundance fungi” based on the PA-DMA method.

LEfSe analysis (LDA > 4.5) identified rhizosphere-specific biomarkers (Figure 4C and Supplementary Table S8): WFt featured o_Hypocreales, f_Nectriaceae, g_Ilyonectria; HFt featured e_Dothideomycetes, o_Pleosporales, s_Mortierella_elongata; PFt featured s_Codinaea_acaiae, g_Codinaea, o_Chaetosphaerales, f_Chaetosphaeriaceae. These findings provide key clues for revealing the functional traits and ecological mechanisms of microbial communities across groups.

### 3.5 Screening of IRM based on companion plants

**FIGURE 5:**
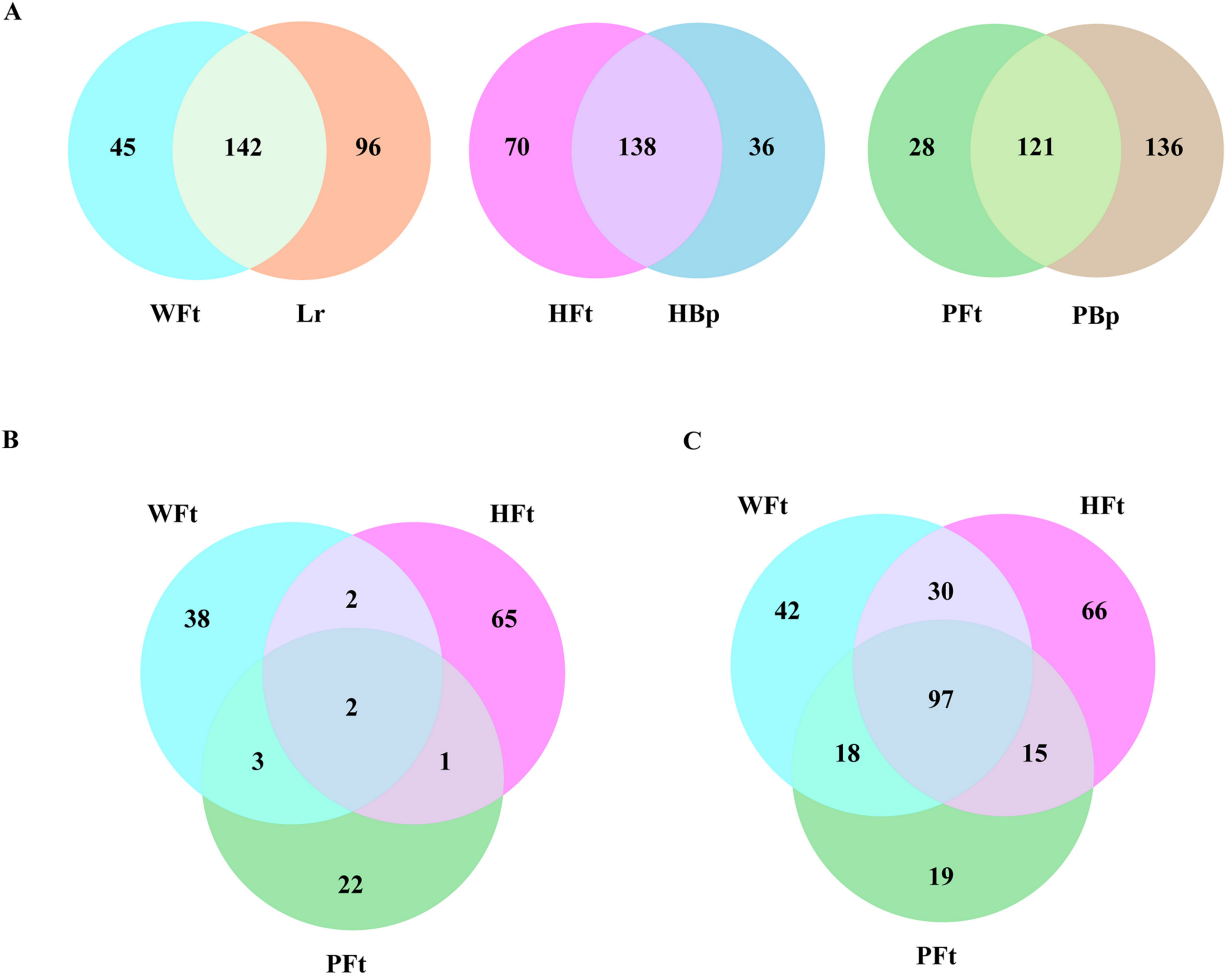
Venn diagram of the distribution of rhizospheric fungal genera in *F. thunbergii* and its companion plants. **(A)** Venn diagram of the distribution of fungal genera in *F. thunbergii* and its companion plants across different cultivation sites. **(B)** Venn diagram of the distribution of unique fungal genera in *F. thunbergii* compared with companion plants across three sites. **(C)** Venn diagram of the distribution of fungal genera in *F. thunbergii* across three sites with direct comparison.

Beta diversity analysis of *F. thunbergii* and companion plants revealed overlapping rhizosphere fungal communities, indicating that shared microbial taxa coexist with host-specific differentiation within the same production area (Supplementary Figure S3). Analysis based on presence/absence of genus-level relative abundance revealed that WFt vs. companion plants had 45 unique fungal genera; HFt vs. companion plants had 70 unique fungal genera; and PFt vs. companion plants had 28 unique fungal genera (Figure 5A). Further analysis revealed that only *Clavatospora* and *Pestalotiopsis* were present at all three *F. thunbergii* sites but were undetected in any companion plant rhizosphere (Figure 5B). Moreover, a direct comparison of three *F. thunbergii* sites without removing shared microbes revealed 97 common fungal genera (Figure 5C), indicating that most were nonspecific fungal genera. The *Pestalotiopsis* relative abundances were 0.14834% (WFt), 0.00106% (HFt), and 0.00042% (PFt), with two cultivated sites below the minimum abundance threshold (0.005%). The *Clavatospora* abundances were 0.1147% (WFt), 0.0196% (HFt), and 0.0087% (PFt), all of which met the threshold requirements. Therefore, *Clavatospora* was defined as the persistently conserved symbiotic fungal genus throughout *F. thunbergii* domestication, serving as the IRM for novel screening methods.

### 3.6 Recalculation of relative community quantity based on IRM relative abundance and differential microorganism analysis

**FIGURE 6:**
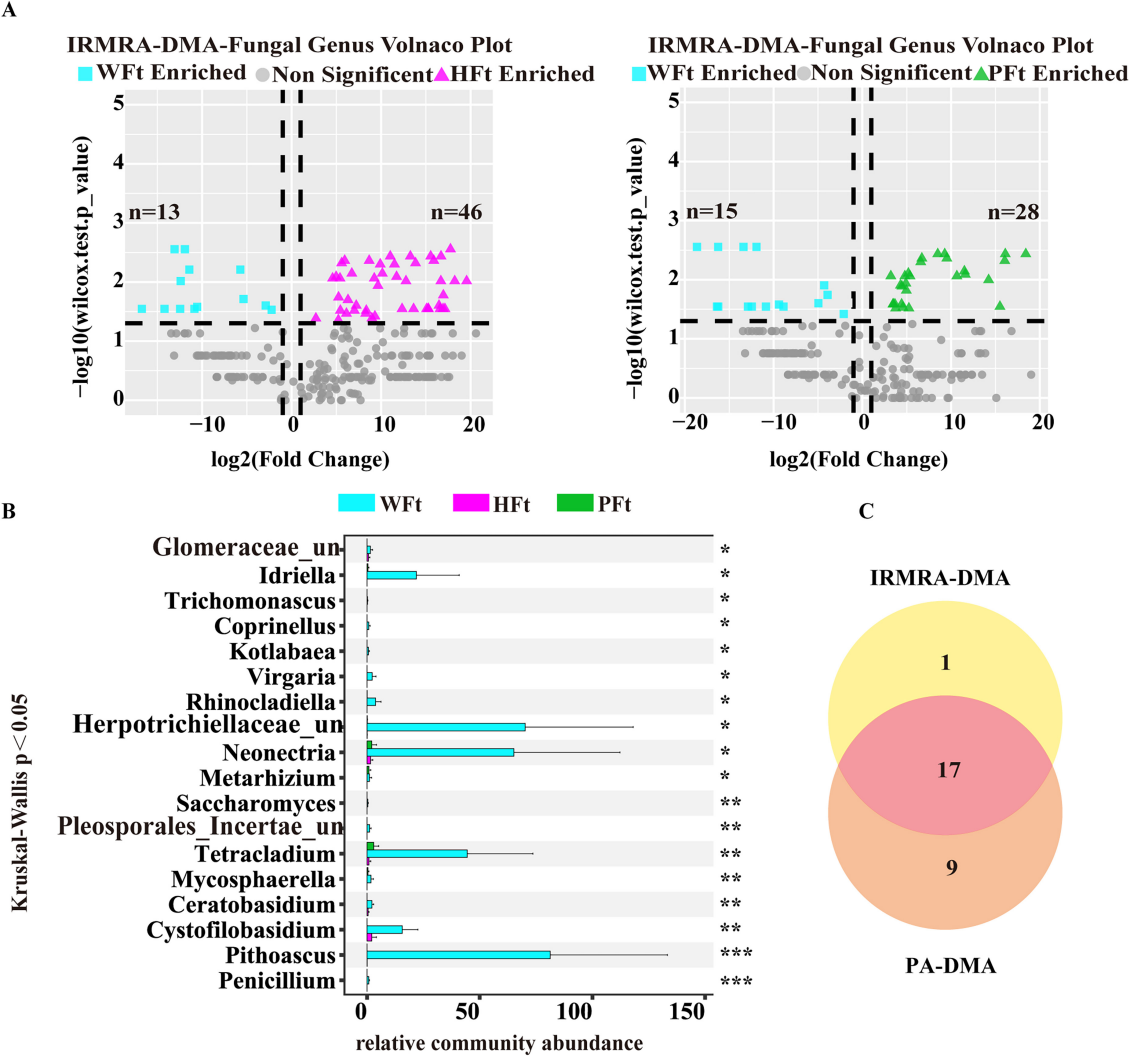
Identification of differential fungal genera via the IRMRA-DMA method and comparison with PA-DMA method. **(A)** Volcano plot showing the differences in fungal genera between wild and cultivated *F. thunbergii* from two areas (Wilcoxon test, *P* < 0.05). **(B)** Analysis of differential fungal genera between wild and cultivated *F. thunbergii* after recalculating abundance based on the IRMRA-DMA method, performed using the Kruskal-Wallis test (*P < 0.05, **P < 0.01, ***P < 0.001, “un” indicates “unclassified”). **(C)** Venn diagram of differential microorganisms screened by the IRMRA-DMA method and PA-DMA method.

The relative community quantity of other fungal genera in the three locations was recalculated using the abundance of *Clavatospora* in *F. thunbergii* as the internal control. Notably, the average abundances of *Clavatospora* in WFt, HFt, and PFt were 0.1147, 0.0196, and 0.0087%, respectively, indicating that its abundance in the two cultivated *F. thunbergii* locations was significantly lower than that in the wild *F. thunbergii*. Thus, its use as an internal control increased the amount of the rhizosphere microbial community in cultivated *F. thunbergii.* Specifically, the PA-DMA method converts the sum of rhizosphere microbiome abundances in each experimental group to 1 (100%), whereas the IRMRA-DMA method used in this study yields mean abundance sums of 37.37 for WFt, 520.62 for HFt, and 679.56 for PFt. This finding indicates that the IRMRA-DMA method overcomes the constraint of microbiome data closure and alleviates the “compression effect” exerted by microorganisms with excessively high absolute abundances on the relative abundances of other microorganisms. The fungal genus volcano plot based on the IRMRA-DMA method (Figure 6A) revealed that, compared with HFt, WFt had 13 significantly upregulated fungal genera and 46 significantly downregulated fungal genera (Supplementary Table S9; Wilcoxon test, *P* < 0.05), with the upregulated fungal genera accounting for 22.03% of the total differential fungal taxa. Compared with PFt, WFt had 15 significantly upregulated fungal genera and 28 downregulated considerably fungal genera (Supplementary Table S10; Wilcoxon test, *P* < 0.05), with the upregulated fungal genera accounting for 34.88% of the total differential fungal taxa. Compared with that of the PA-DMA method, the proportion of downregulated fungal genera significantly increased, indicating that the IRMRA-DMA method corrected some of the effects of “closure” on relative abundance. Further analysis of rhizosphere fungal genus differences among the three locations via the Kruskal-Wallis test revealed a total of 80 significantly different genera (Supplementary Table S11). Among them, 18 fungal genera presented significantly greater relative abundances in the wild samples than in the samples from the two cultivated locations (Figure 6B). Thus, these 18 genera were defined as “wild-enriched high-abundance fungi” based on the IRMRA-DMA method.

### 3.7 Comparative analysis of IRMRA-DMA method and PA-DMA methods

The differential fungal genera separately screened via the IRMRA-DMA method and the PA-DMA method were analyzed and summarized (Figure 6C and Supplementary Table S12). Seventeen common differential fungal genera were screened via both the IRMRA-DMA method and the PA-DMA method. The IRMRA-DMA method screened out one new differential fungal genus, *Trichomonascus*, which was excluded from the PA-DMA method screening because of its low abundance. This finding demonstrated that the low-abundance IRM highlighted some differential fungal genera with relatively low expression levels, whose low abundance might have been excluded from the calculation of the Kruskal-Wallis test. The IRMRA-DMA method did not detect 9 fungal genera screened by the PA-DMA method. The reason is that these genera exhibited “suppressed” relative abundance due to the excessively high abundance of other genera, thus appearing as significant differences, which were not genuine. The correction effect of the IRMRA-DMA method eliminated this outcome. The common differential fungal genera included *Penicillium*, *Pithoascus*, and *Virgaria*, among which *Penicillium* is a well-known plant probiotic with significant application potential in areas such as plant growth promotion and pathogen antagonism. Some fungi in the genera *Pithoascus* and *Virgaria* are saprophytes that degrade plant residues in soil, potentially participating in ecosystem nutrient cycling and enhancing soil fertility. This may be a potential reason for the better resistance and quality of wild *F. thunbergii*. The above results indicate that the IRMRA-DMA method can effectively filter out false-positive differential genera arising from data noise. While retaining biologically significant differences among fungal genera, it enhances the precision and reliability of screening for rhizosphere fungal genus biomarkers. Furthermore, it can reveal some new differential microorganisms that exhibit dual efficacy in the process of mining differential microorganisms, providing a new approach for screening differential microorganisms.

### 3.8 Isolation and functional analysis of differential microbial taxa in the rhizosphere of wild *F. thunbergii*

A total of 165 rhizosphere fungal strains belonging to 30 genera were isolated from the rhizosphere of wild *F. thunbergii*. Among them, *Fusarium* had the most significant number of strains, reaching 38; followed by *Trichoderma* with 27 strains; *Cunninghamella* with 23 strains; Alternaria with 18 strains; and the number of strains in the remaining 25 genera was ≤ 10 (Supplementary Figure S4). Guided by the previous PA-DMA and IRMRA-DMA analyses, which identified *Penicillium* as a core differential fungal genus, we focused on two specific *Penicillium* strains isolated in this study, designated as WFt-032 and WFt-056. To investigate their potential role in disease suppression, we performed a Spearman correlation analysis between *Penicillium* and the two major *F. thunbergii* pathogenic genera (*Fusarium* and *Alternaria*) using the corrected absolute abundance data (Supplementary Table S11). The results revealed significant negative correlations between Penicillium and both Fusarium (*R* = −0.51, *P* = 0.031) and Alternaria (*R* = −0.53, *P* = 0.024). These findings suggest a potential antagonistic relationship, indicating that Penicillium may play a role in inhibiting these pathogens in the *F. thunbergii* rhizosphere.

**FIGURE 7:**
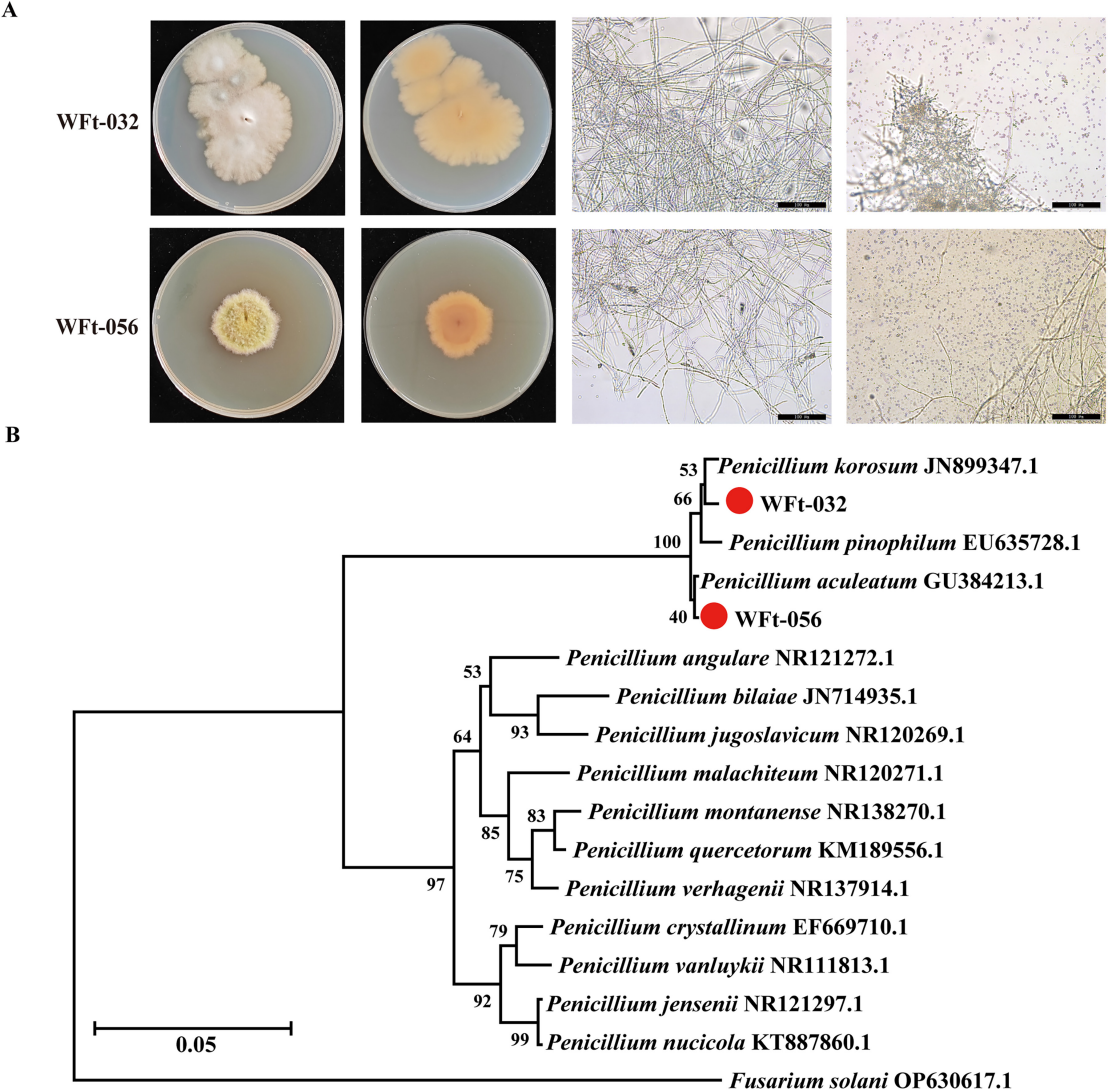
Isolation and identification of rhizospheric fungal taxa from wild *F. thunbergii*. **(A)** Colonial morphology of two *Penicillium* strains. **(B)** Phylogenetic tree analysis of isolated strains’ ITS sequences with published *Penicillium* ITS sequences.

Under cultivation conditions at 28°C on PDA medium, the colony morphology of WFt-032 was irregular, with white mycelium and no exudate; the colony of WFt-056 was irregularly round, with yellowish-green mycelium and light yellowish exudate visible on the medium surface (Figure 7A). Phylogenetic analysis of the ITS2 sequences of the strains revealed that WFt-032 had the highest sequence similarity to *Penicillium korosum* and that WFt-056 had the highest sequence similarity to *Penicillium aculeatum*. Combining the colony morphological characteristics and molecular identification results, the isolated strain WFt-032 was identified as *Penicillium korosum* (NCBI accession number PV938099), and WFt-056 was identified as *Penicillium aculeatum* (NCBI accession number PV938100) (Figure 7B).

### 3.9 Analysis of the plant growth promotion and antimicrobial capabilities of WFt-032 and WFt-056

**FIGURE 8:**
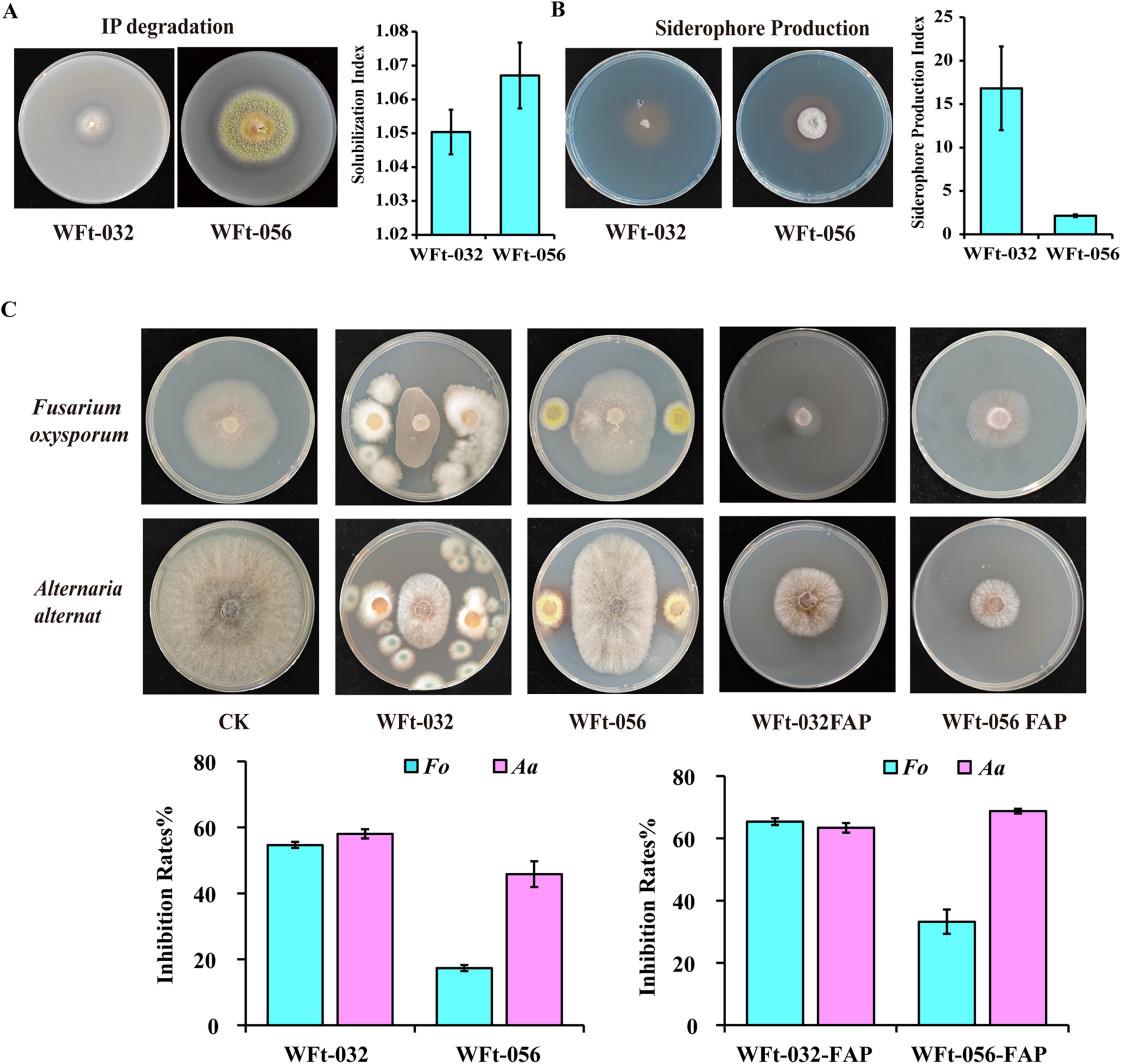
Analysis of the plant growth-promoting and antibacterial abilities of WFt-032 and WFt-056. **(A)** Detection of their phosphate-solubilizing abilities. **(B)** Detection of their siderophore-producing abilities. **(C)** Antagonistic effects and inhibition rates of WFt-032 and WFt-056 against the primary disease pathogens of *F. thunbergii*, including the plate confrontation method and the fermentation filtrate method. FAP, Filtrate antimicrobial potential.

To evaluate the plant growth-promoting capabilities of WFt-032 and WFt-056, their inorganic phosphate solubilization and siderophore-producing capabilities were analyzed. After 5 days of culture in inorganic phosphate media, transparent phosphate solubilization zones formed around both strains WFt-032 and WFt-056. The SI value of WFt-032 was 1.05, and that of WFt-056 was 1.07, indicating that both strains possess phosphate solubilization capabilities, with WFt-056 exhibiting stronger capabilities (Figure 8A). After 5 days of culture in CAS media, orange halos appeared around both strains, indicating siderophore production. The SPI value of WFt-032 was 16.8, and that of WFt-056 was 2.14, demonstrating that both strains possess siderophore-producing capability, with WFt-032 exhibiting greater capability than WFt-056 (Figure 8B).

*F. oxysporum* (E-8, NCBI accession number PV938101) and *A. tenuissima* (E-29, NCBI accession number PV938102) were isolated from diseased bulbs of *F. thunbergii* (Supplementary Figure S5). The dual-culture antagonism results revealed that the inhibition rate of WFt-032 against *F. oxysporum* was 54.67%, and that against *A. tenuissima* was 58.02%. For WFt-056, the inhibition rate against *F. oxysporum* was 17.3%, and that against *A. tenuissima* was 45.81%. The fermentation filtrate assay results revealed that the inhibition rate of the WFt-032 filtrate against *F. oxysporum* reached 65.35%, and that against *A. tenuissima* reached 63.37%. For the WFt-056 filtrate, the inhibition rate against *F. oxysporum* was 33.21%, and that against *A. tenuissima* was 45.81% (Figure 8C). The above experimental results indicate that, compared with the blank control, both WFt-032 and WFt-056 effectively inhibited the growth of *F. oxysporum* and *A. tenuissima*.

### 3.10 Effects of WFt-032 and WFt-056 on bulb disease resistance and expression of disease resistance-related genes in *F. thunbergii*

**FIGURE 9:**
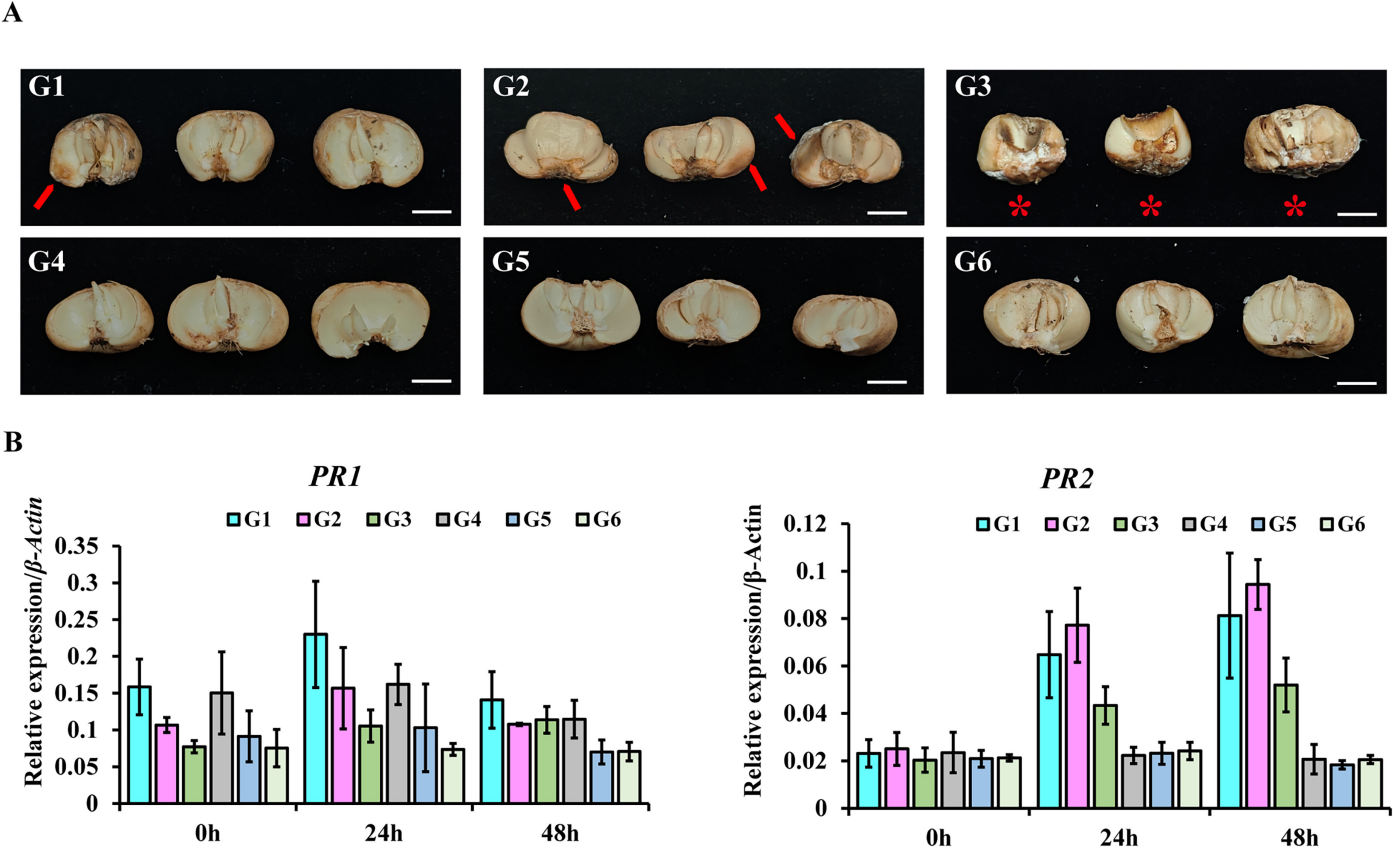
Effects of WFt-032 and WFt-056 on bulb disease resistance and expression of disease resistance-related genes in *F. thunbergii*. **(A)** The fermentation filtrates of WFt-032 and WFt-056 delayed the decay of *F. thunbergii* bulb. Arrows indicate localized decay areas; * marks completely decayed samples due to overall tissue discoloration and softening. Scale bar = 1 cm. **(B)** Effects of WFt-032 and WFt-056 fermentation filtrates on the expression of resistance-related genes in *F. thunbergii*, data are presented as mean ± standard deviation (*n* = 3).

To evaluate the effects of strains WFt-032 and WFt-056 on the disease resistance of *F. thunbergii* bulbs, assessments of disease resistance and detection of expression levels of disease resistance-related genes were conducted on bulbs treated with the fermentation filtrates of WFt-032 and WFt-056. The results showed that the fermentation filtrates of WFt-032 and WFt-056 significantly alleviated bulb rot. In contrast to the almost complete rot observed in the control group G3, bulbs treated with WFt-032 and WFt-056 fermentation filtrates only exhibited mild rot, indicating that these treatments delayed the progression of bulb rot (Figure 9A). Moreover, the disease resistance effect of WFt-032 fermentation filtrate was superior to that of WFt-056.

Disease resistance-related genes displayed different expression patterns. Group G1 showed the strongest expression-inducing effect on *PR1*; its inducing effect on *PR2* was weaker than that of G2, but the expression levels at corresponding time points were all higher than those in its control group G4. Group G2 induced the expression of *PR1* and PR2, with levels higher than those in its control group G5; notably, PR2 expression at 48 h in G2 was 5.15-fold that in G5. Comparison between group G3 and its control group, G6, showed that treatment with pathogens alone could slightly increase the expression of resistance genes, but the magnitude of the increase was lower than that in G1 and G2. At the gene level, PR2 expression in pathogen-inoculated groups gradually increased over time, while PR1 reached its highest expression at 24 h and then decreased (Figure 9B). These results indicate that the fermentation filtrates of WFt-032 and WFt-056 can enhance and induce disease resistance in *F. thunbergii*, with WFt-032 exhibiting a more significant effect.

## 4 Discussion

### 4.1 Differential microorganism screening approach based on IRM

Plants have gradually developed close symbiotic relationships with specific microorganisms during domestication (Deng et al., 2023; Yu et al., 2024; Wang D. et al., 2025; Wang S. et al., 2025). High-throughput amplicon sequencing of the fungal internal transcribed spacer (ITS) region has become the most essential means for mining key microorganisms in the plant rhizosphere (Edwards et al., 2015; Müller et al., 2016). However, traditional differential microorganism screening methods suffer from significant limitations because they rely on relative abundance calculations. The implicit assumption of “constant total microbial load” contradicts the dynamic nature of soil environments: abiotic factors such as pH, nutrient gradients, and temperature/humidity can significantly alter total microbial biomass (StämmLer et al., 2016), leading to spurious relative abundance differences for taxa with the same absolute abundance after normalization (Weiss et al., 2017). More critically, the compositional closure characteristic of microbiome data means that a change in the relative abundance of one taxon inevitably causes compensatory fluctuations in others, leading to false-positive and false-negative results.

Spiking internal controls is a good strategy for quantifying microbial load. In recent years, methods such as spiking known sequences (Smets et al., 2016; Lin et al., 2019), spiking cultures not present in the community (StämmLer et al., 2016), using synthetic 16S internal controls (Tourlousse et al., 2017), and employing combinations of synthetic internal controls for 16S, 18S, and ITS (Tkacz et al., 2018) have been developed for quantitative analysis of the microbiome. However, these spike-in-based methods also have limitations. For instance, universal microbial genes such as the 16S rRNA gene and ITS share high sequence similarity with sequences in plant genomes, making it impossible to detect microbial load by comparing microbial and plant genes. Therefore, quantitative detection of microbiome abundance remains a significant challenge. To address these bottlenecks, this study proposes an IRM screening system based on the companion plants of *F. thunbergii*. Its core breakthroughs lie in the following three aspects: (i) The ecological rationality of the internal control that is based on companion plants, fungi that stably coexist long-term in the rhizosphere of *F. thunbergii* are screened as the internal control metric. This avoids the arbitrariness of “artificially setting a reference benchmark” in traditional methods. (ii) The absolute abundance correction mechanism; since companion plants and target plants share geographical adaptability and niche conservatism, the abundance of the IRM genuinely reflects environmental impacts on total microbial load. Using the IRM as a baseline to recalibrate the abundance of other microorganisms effectively decouples the compositional closure constraint. For example, when environmental stress decreases total microbial load, the IRM abundance decreases synchronously, keeping the corrected ratio of non-differential taxa stable while allowing truly differential taxa to breach significance thresholds. (iii) A significant reduction in redundancy; after integration with traditional relative abundance analysis, the redundancy in screening is reduced by more than 30% by filtering out “total load fluctuation noise” and avoiding misjudgments caused by inter-sample biomass differences. This method provides a new perspective for cross-scale integration in microbiome research. In agricultural ecosystems, companion plants are not only ecological partners of the target crop; their rhizosphere microbiomes can also serve as “environmental sensors,” providing a calibration reference for resolving functional differences in the target crop’s microbiome. Selecting *Lycoris radiata* and *Brassica pekinensis* as background species not only effectively eliminates background interference caused by soil physicochemical heterogeneity but also enhances the detection sensitivity of host-specific microbial signals because of their relatively distant phylogenetic relationships with *F. thunbergii*. The advantages of this system lie not only in improved statistical power but also in its closed-loop ecological logic design: the IRM acts as an “ecological yardstick.” Correction based on this benchmark, by anchoring a stable reference value, transforms relative abundance into a “semiquantitative abundance” closer to absolute abundance. This approach transforms host-microbe coevolutionary relationships into quantifiable correction factors, providing a new paradigm for analyzing the absolute enrichment patterns of functional microorganisms.

### 4.2 Isolation and application of key microbial taxa in the rhizosphere of wild *F. thunbergii*

The process of plant domestication significantly affected the plant rhizosphere microbial community. In contemporary agricultural production systems, intensive cultivation practices such as high-density planting, excessive water and fertilizer management, and pesticide use are prevalent to maximize crop quality and yield. This phenomenon has led to the emergence of “domestication syndrome” in numerous plants during long-term domestication and breeding improvement processes. For example, continuous domestication selection and years of breeding have significantly reduced the genetic diversity of modern grape varieties, accompanied by the loss of resistance genes, markedly increasing their susceptibility to pests, diseases, and abiotic stresses (Guo et al., 2025). Similarly, during tomato domestication, specific alterations occurred in the SlMYB78-regulated phenolamide and salicylic acid biosynthesis gene cluster, resulting in a significant decline in plant disease resistance (Cao P. et al., 2025). In the cultivation of *F. thunbergii*, this syndrome is particularly pronounced, manifesting as significantly weakened disease resistance, frequent outbreaks of soil-borne diseases, and even complete crop failure in severely affected plots, resulting in substantial economic losses for growers.

The rhizosphere microbiota plays a central role in enhancing plant environmental adaptability and productivity, particularly by performing irreplaceable tasks in response to biotic and abiotic stresses (Liu H. et al., 2020; Liu et al., 2025 Angot et al., 2025). Research indicates that a complex bidirectional interaction network exists between plants and rhizosphere microorganisms: some microbes act as potential pathogens that cause plant diseases or even establish disease cycles, leading to plant death (Bartoli et al., 2018; Redkar et al., 2022; Tamburrini et al., 2024), whereas beneficial microbes enhance disease resistance through mechanisms such as promoting plant growth and activating immune responses (Akram et al., 2016; Pérez-Jaramillo et al., 2016; Fan et al., 2024).

Based on the above theory, Raaijmakers and Kiers proposed the “Plant Microbiome Rewilding” hypothesis in 2022 (Raaijmakers and Kiers, 2022), suggesting that restoring the abundance of key microbial taxa from the rhizosphere of a crop plant’s wild ancestors can reshape the rhizosphere microbial community structure, thereby significantly improving the growth performance and disease resistance of cultivated varieties. This provides an innovative approach to addressing “domestication syndrome” and the obstacles to continuous cropping. For research on *F. thunbergii*, the key to achieving plant microbiome rewilding lies in systematically mining the core companion microbiota lost during domestication, deeply analyzing its functions, and reintroducing it into the rhizosphere of cultivated plants.

The results of our analysis of rhizosphere fungi in wild and cultivated *F. thunbergii* align with expectations: based on alpha and beta diversity analyses, significant differences exist in species richness and community composition between the rhizosphere fungal communities of wild-type and cultivated-type *F. thunbergii*. Furthermore, rhizosphere fungal correlation network analysis revealed that the rhizosphere fungal network of wild *F. thunbergii* exhibited increased complexity and modular dispersion. This structural characteristic may represent an essential strategy for the host plant to maintain ecological balance and cope with complex stresses in the natural environment (Faust and Raes, 2012; Coyte et al., 2015), and it also aligns with the general trend of microbial community simplification during plant domestication—compared with modern cultivated crop varieties, crop wild ancestors can recruit functionally distinct microbiomes (Wang C. et al., 2024). Isolating and utilizing these microorganisms is of profound significance for improving the disease resistance of cultivated varieties.

### 4.3 Analysis of the plant growth promotion and antimicrobial effects of *Penicillium*

In the field of agricultural production, the use of microorganisms for the biological control of plant diseases as a green, sustainable control strategy is gradually becoming a research and application hotspot (Alabouvette et al., 2009; Gajbhiye et al., 2010; Du et al., 2025). Based on fungal genus-level analysis of the rhizosphere fungal taxa of *F. thunbergii* and screening for differential microorganisms, this study successfully isolated two *Penicillium* strains from the rhizosphere soil of wild *F. thunbergii*. *Penicillium*, a common plant probiotic, has significant potential for applications in plant growth promotion and pathogen antagonism. Existing studies indicate that the *Penicillium* strain 47 M-1 isolated from tobacco rhizosphere soil has an 81.3% inhibition rate of *Fusarium oxysporum* mycelial growth and achieves 50% field control efficiency against sesame *Fusarium* (Zhao et al., 2021). Doilom et al. also reported that *Penicillium* fungi possess inorganic phosphate-solubilizing capabilities (Doilom et al., 2020); *P. bilaiae* MA-267 isolated from *Lumnitzera racemosa* rhizosphere soil secretes penicibilaenes A and B, which have significant inhibitory effects on pathogenic fungi such as *Colletotrichum gloeosporioides* (Meng et al., 2014).

The *Penicillium* strains WFt-032 and WFt-056 isolated in this study significantly inhibited the mycelial growth of *F. thunbergii* bulb rot disease *F. oxysporum* and the black spot pathogen *Alternaria tenuissima*, demonstrating broad-spectrum antimicrobial activity. Soil microorganisms can promote crop growth by increasing the capacity to solubilize inorganic phosphate (Brazhnikova et al., 2022; Huang et al., 2025). The results of this study are consistent with the above description; both WFt-032 and WFt-056 exhibit the ability to efficiently solubilize insoluble inorganic phosphorus. Iron is an essential micronutrient for plant growth and is involved in key physiological processes such as photosynthesis, respiration, and nitrogen metabolism. However, the low solubility of Fe^3+^ in aerobic environments significantly reduces the efficiency of plant iron absorption. Siderophores, as low-molecular-weight compounds that efficiently chelate Fe^3+^, play crucial roles in plant iron acquisition. Soil microorganisms can increase Fe^3+^ bioavailability through siderophore secretion (Zhang et al., 2024; Chen et al., 2025). After 5 days of culture on solid CAS media, distinct orange halos formed around colonies of both WFt-032 and WFt-056, confirming their siderophore production capability and indicating their potential to promote *F. thunbergii* growth.

Further research revealed that in *F. thunbergii* bulbs treated with fermentation filtrates of WFt-032 and WFt-056, the expression levels of systemic resistance-related genes (e.g., the PR gene family) were significantly increased, indicating that these strains enhance disease resistance by activating plant defense responses. However, their true disease resistance mechanisms still require further in-depth research. In summary, the isolated *Penicillium* strains WFt-032 and WFt-056 have significant potential for promoting *F. thunbergii* growth and disease control, demonstrating broad prospects for biological control of diseases in medicinal plants.

### 4.4 Limitations and future work

However, the IRM-corrected differential microbial analysis (IRMRA-DMA) method still faces challenges: a core limitation is that the regional abundance stability of IRMs has not yet been systematically validated through multi-omics integration analysis and absolute quantification techniques. In future research, we will focus on multi-omics integration and absolute quantification techniques to systematically carry out method validation and optimization work: First, integrate multi-omics data including genomics, transcriptomics, and metabolomics to thoroughly analyze the genetic background, gene expression patterns, and functional metabolite characteristics of IRMs in the rhizosphere of *Fritillaria thunbergii* across different regions, clarify the correlation mechanism between IRM abundance and environmental factors, and explain its regional adaptability at the molecular level. Second, adopt high-precision absolute quantification techniques such as digital PCR and flow cytometry to accurately determine the absolute abundance of IRMs in regions with different climate zones and soil types (e.g., acidic red soil, neutral loam), establish a standard for IRM abundance fluctuation thresholds, and verify the calibration reliability of the IRMRA-DMA method under different environmental conditions. Third, further optimize the IRM screening criteria based on multi-omics validation and absolute quantification to ensure the selected IRMs exhibit broad regional stability. Moreover, future research can expand the IRM screening criteria and processing methods for highly sparse data and explore the generalizability of this method across multi-crop systems. Based on the above research, the robustness and generalizability of the IRMRA-DMA method can be significantly improved, providing more reliable technical support for cross-regional and multi-species microbiome differential analysis and promoting the in-depth development of microbiome function analysis research.

## 5 Conclusion

**FIGURE 10:**
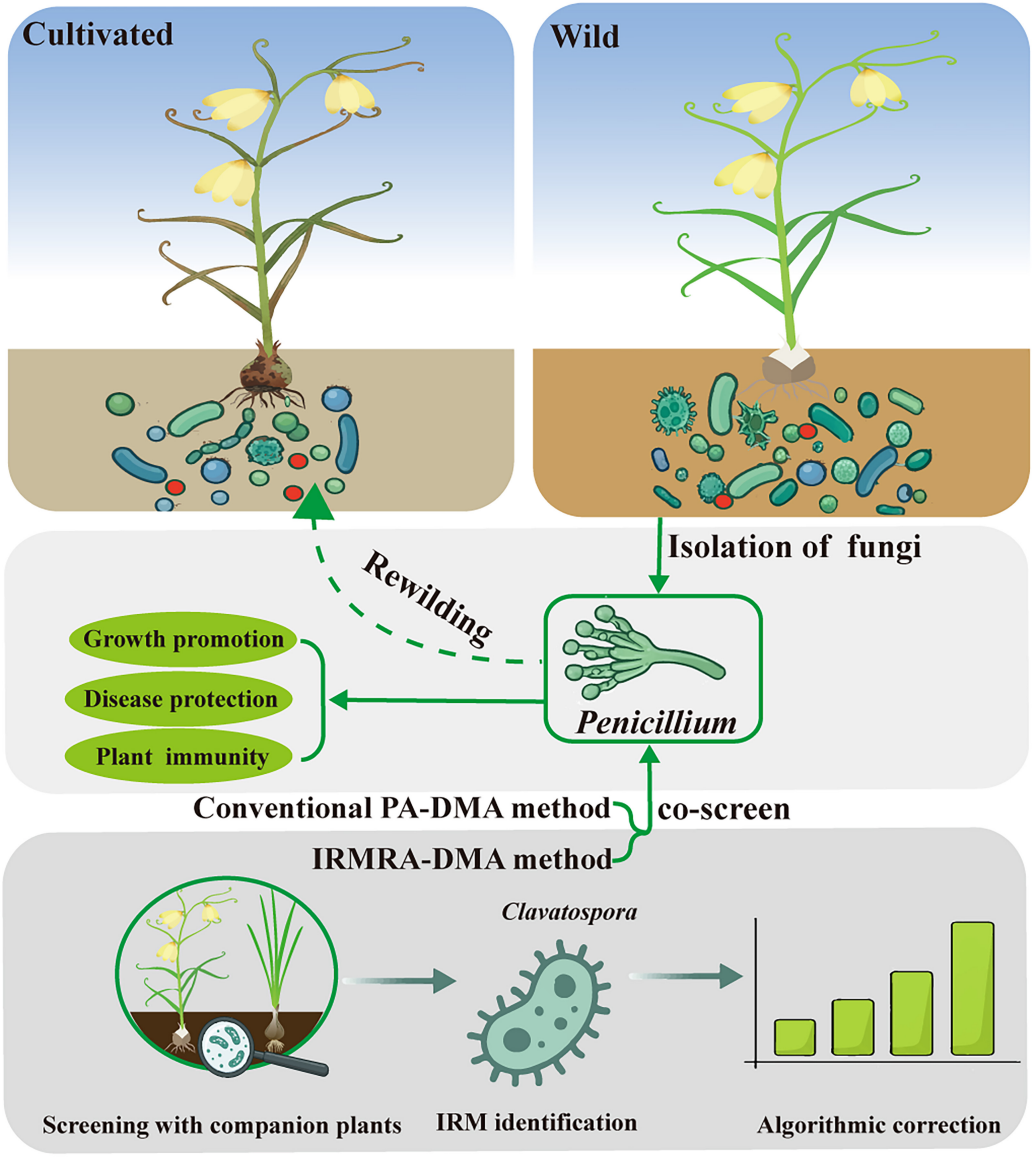
A dual-methodology framework, integrating conventional PA-DMA and optimized IRMRA-DMA, was employed to co-screen and isolate the core functional candidate fungus *Penicillium* and analyze its functions; the dashed line indicates the prospect of *Penicillium* rewilding. (Bottom) Detailed mechanistic workflow of the IRMRA-DMA method, including companion plant screening, IRM identification and algorithmic correction for enhanced selection accuracy.

This study reveals that long-term artificial cultivation significantly alters the rhizosphere fungal community of *F. thunbergii*, characterized by reduced biodiversity and a shift in dominant taxa from beneficial symbionts to potential pathogens. To address the limitations of standard relative abundance analysis, we identified *Clavatospora* as a host-specific IRM that remains stable across diverse habitats. By establishing the IRMRA-DMA method, we successfully corrected the data closure bias inherent in compositional data, enabling more accurate quantification of absolute abundance variations. Crucially, this methodological innovation highlighted *Penicillium* as a key wild-enriched genus in the *F. thunbergii* rhizosphere. Subsequent isolation and functional validation confirmed its dual roles in plant growth promotion and disease suppression (Figure 10). Based on these findings, we recommend avoiding sole reliance on relative abundance analysis in rhizosphere research and propose a “microbiome rewilding” strategy: introducing wild-derived functional consortia to restore the functional diversity of cultivated *F. thunbergii.*

## Data Availability

The datasets presented in this study can be found in online repositories. The names of the repository/repositories and accession number(s) can be found in the article/Supplementary material.
