# Supplementary material for: A novel internal reference microorganism-based method reveals wild-enriched Penicillium for enhancing growth and disease resistance in Fritillaria thunbergii
Source: Front Microbiol. 2026 Feb 11;17:1746815. doi: 10.3389/fmicb.2026.1746815 (PMC12932547; doi:10.3389/fmicb.2026.1746815)

**Supplementary Information**

**A novel internal reference microorganism-based method reveals wild-enriched Penicillium for enhancing growth and disease resistance in *Fritillaria thunbergii***

**Contents**

**1. Tables**

**Table S1** Summary of sampling orchards in Zhejiang province of China.

**Table S2** Primers information used for amplicon sequencing in this study.

**Table S3** Amplicon Sequence Variant Feature Table with Annotations.

**Table S4** Topology properties of the networks.

**Table S5** Volcano plot showed the differences in fungal genera between WFt and HFt based on the PA-DMA method.

**Table S6** Volcano plot showed the differences in fungal genera between WFt and PFt based on the PA-DMA method

**Table S7** Significantly different fungal genera identified based on the PA-DMA methoed,

**Table S8** LDA value distribution histogram analysis of species between wild and cultivated *F. thunbergii*

**Table S9:** Volcano plot showed the differences in fungal genera between WFt and HFt after recalculation based on the IRMRA-DMA method.

**Table S10**: Volcano plot showed the differences in fungal genera between WFt and PFt after recalculation based on the IRMRA-DMA method.

**Table S11**: Significantly different fungal genera identified based on the IRMRA-DMA method.

**Table S12**: Summary of high-abundance fungal genus in wild *F. thunbergii* identified by two methods.

**2. Figures**

**Figure. S1** Rhizosphere soil sampling of wild/cultivated *F.* thunbergii and companion plants. **(A)** Geographic locations of wild/cultivated *F. thunbergii* with companion plants. **(B)** Schematic representation of soil collection from target plants.

**Figure. S2** Analysis of differential fungal genera between wild and cultivated *F. thunbergii*. Error bars represent 95% confidence intervals. **(A)**WFt VS HFt. **(B)**WFt VS PFt

**Figure. S3** Non-metric Multidimensional Scaling (NMDS) of the fungal microbiomes in the rhizosphere samples of Fritillaria thunbergii and its associated plants based on Bray-Curtis distance. **(A)**WFt and Lr. **(B)** HFt and HBp. **(C)** PFt and PBp

**Figure. S4** Overview and statistics of isolates of rhizospheric fungal taxa from wild *Fritillaria thunbergii*.

**Figure. S5** Phylogenetic tree analysis of the isolated *Fusarium* strains E-8**(A)** and *Alternaria* strains E-29**(B)** with published strains' sequences

**Figure. S1** Rhizosphere soil sampling of wild/cultivated *F.* thunbergii and companion plants. **(A)** Geographic locations of wild/cultivated *F. thunbergii* with companion plants. **(B)** Schematic representation of soil collection from target plants.


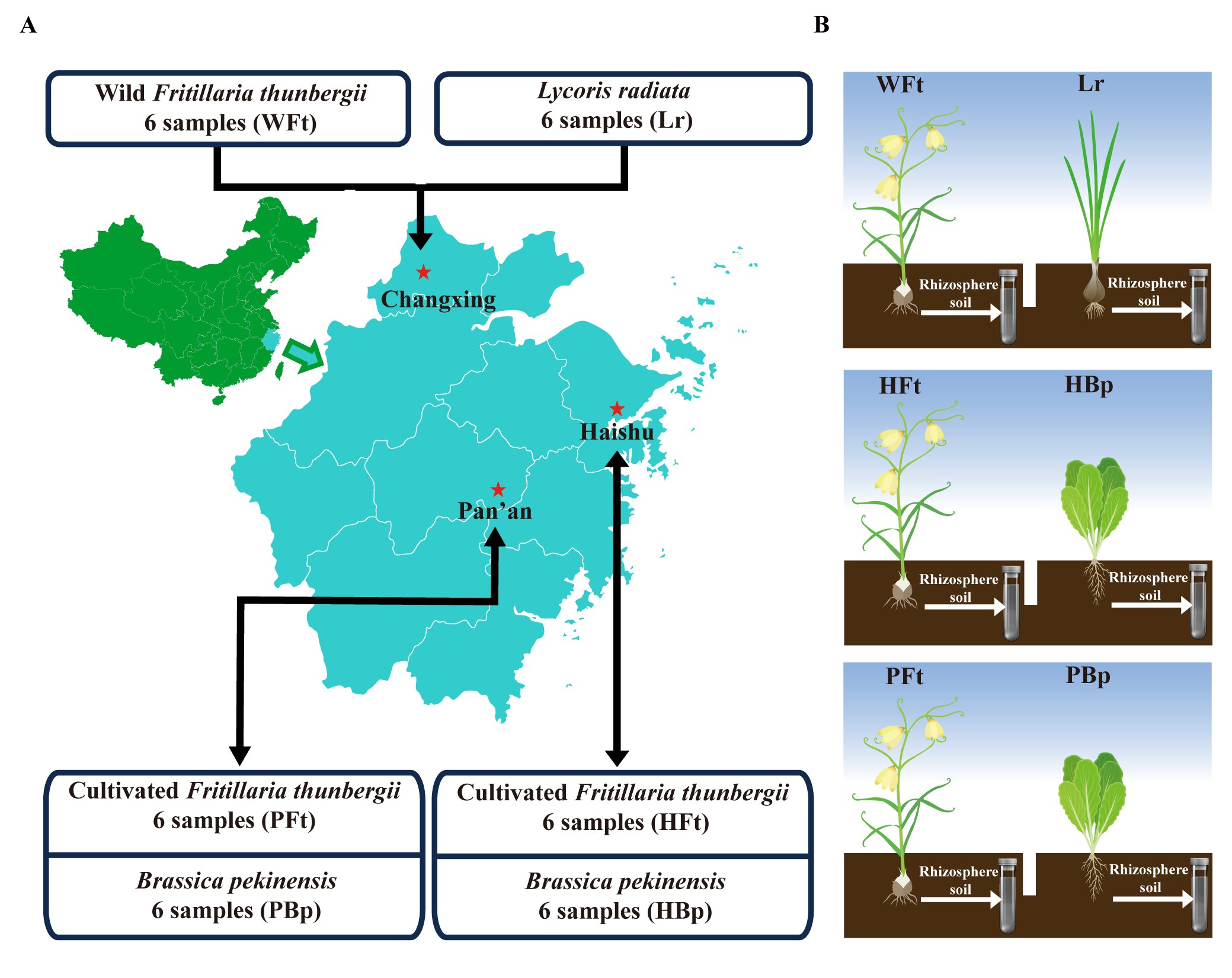


**Figure. S2** Analysis of differential fungal genera between wild and cultivated *F. thunbergii.* Error bars represent 95% confidence intervals. **(A)**WFt VS HFt. **(B)**WFt VS PFt


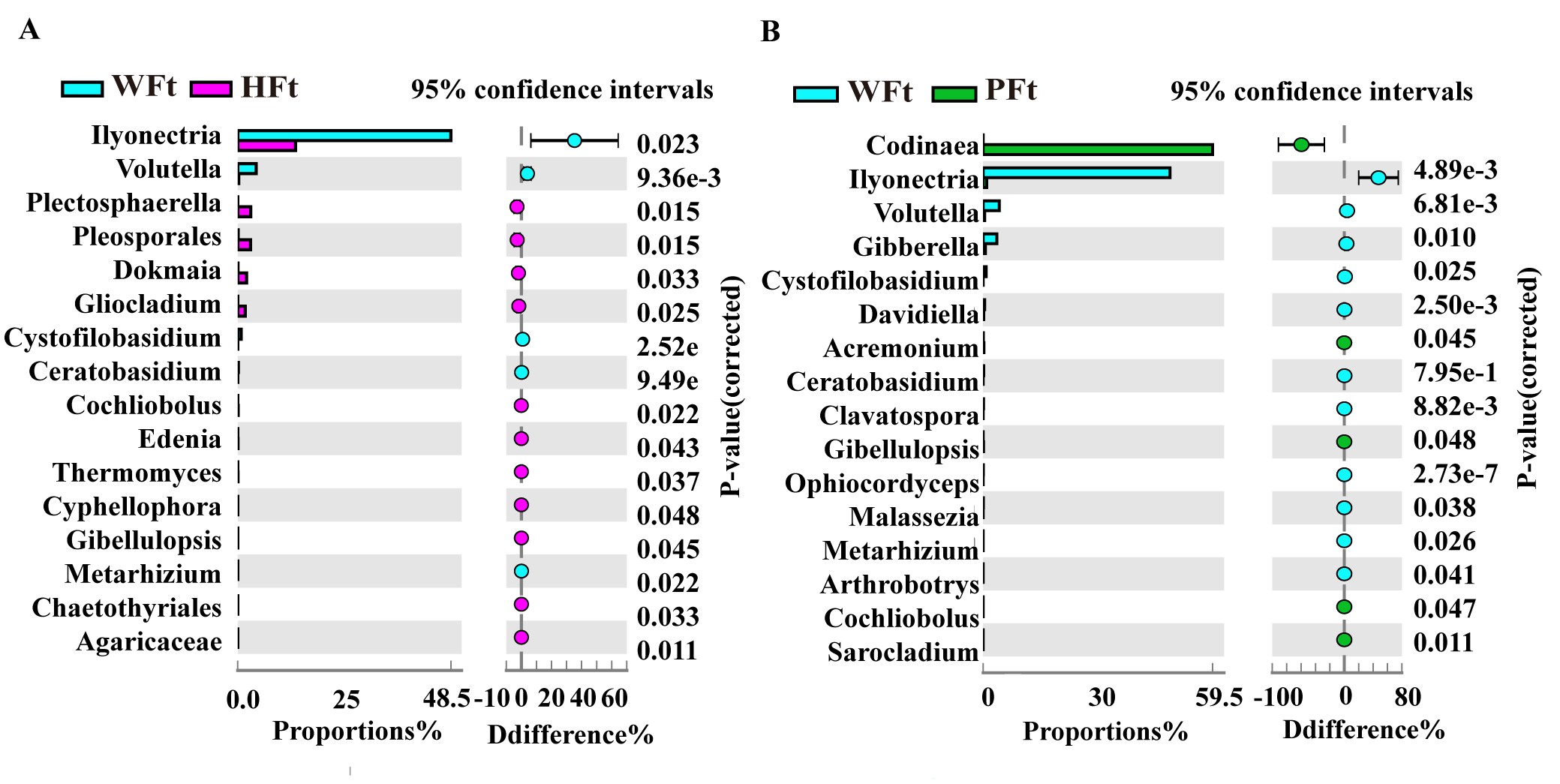


**Figure. S3** Non-metric Multidimensional Scaling (NMDS) of the fungal microbiomes in the rhizosphere samples of *Fritillaria thunbergii* and its associated plants based on Bray–Curtis distance. **(A)**WFt and Lr. **(B)** HFt and HBp. **(C)** PFt and PBp


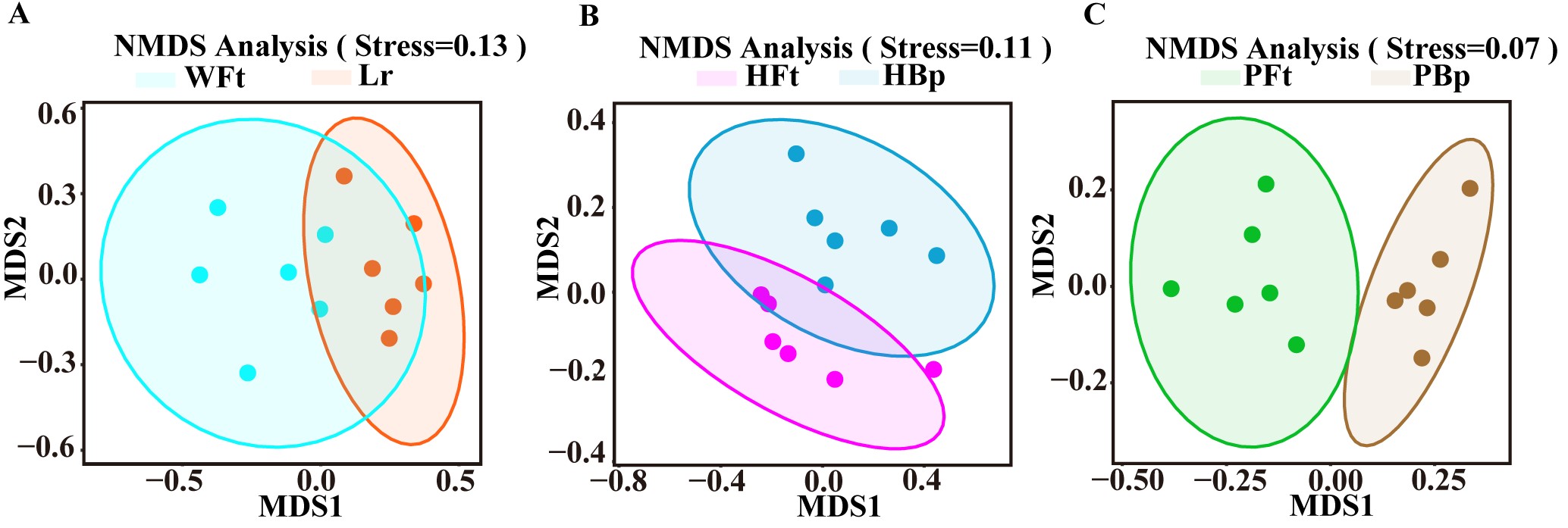


**Figure. S4** Overview and statistics of isolates of rhizospheric fungal taxa from wild *Fritillaria thunbergii*.


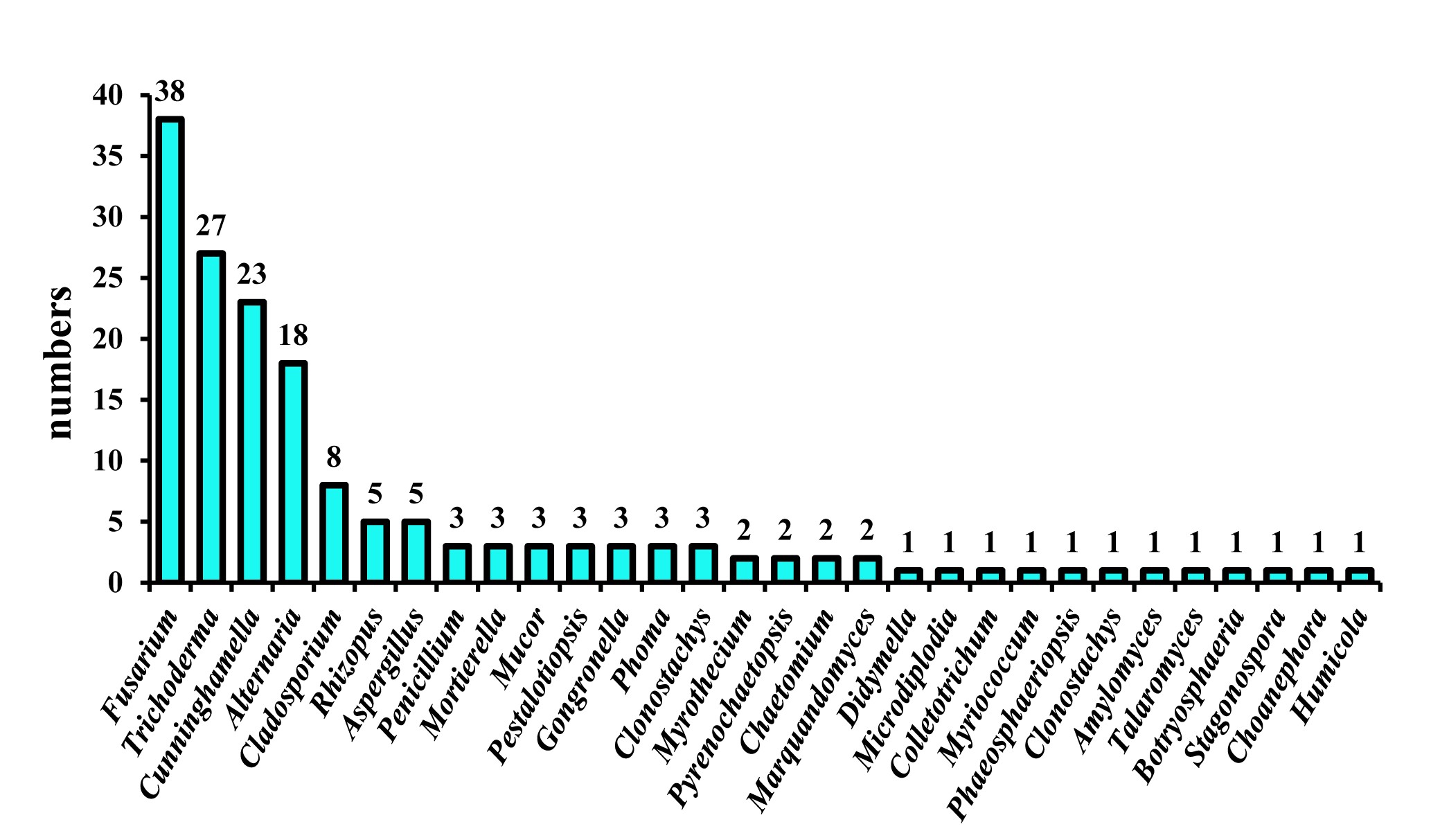


**Figure. S5** Colonial morphology and phylogenetic tree analysis of the isolated *Fusarium* strains E-8 (**A**) and *Alternaria* strains. E-29 (**B**) with published strains sequences.


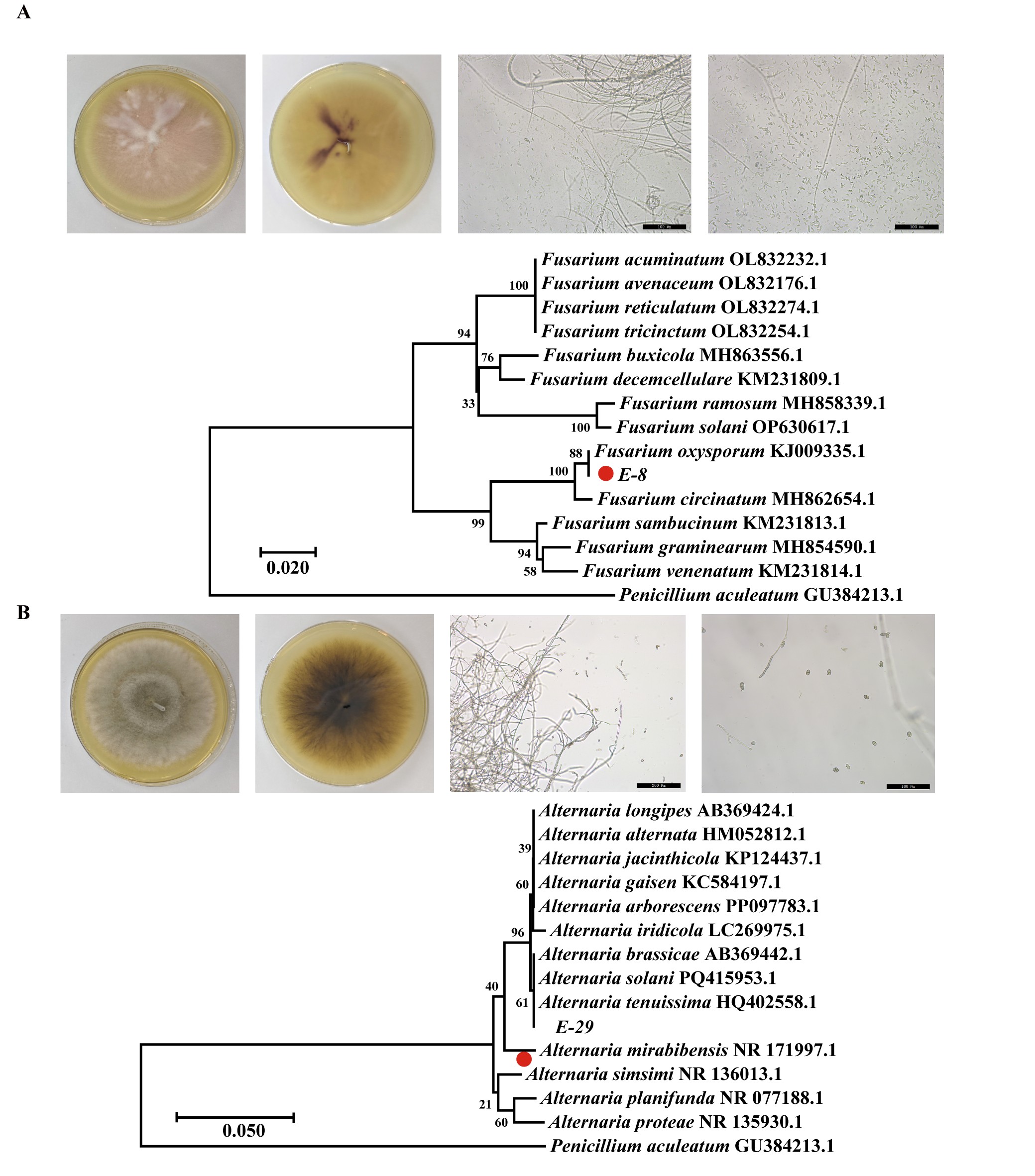

Supplement: Supplementary file 1 [file Data_Sheet_1.docx]
